# Chemical signatures and new drug targets for gametocytocidal drug development

**DOI:** 10.1038/srep03743

**Published:** 2014-01-17

**Authors:** Wei Sun, Takeshi Q. Tanaka, Crystal T. Magle, Wenwei Huang, Noel Southall, Ruili Huang, Seameen J. Dehdashti, John C. McKew, Kim C. Williamson, Wei Zheng

**Affiliations:** 1 National Center for Advancing Translational Sciences, National Institutes of Health, Bethesda, MD 20892, United States; 2 Laboratory of Malaria and Vector Research, National Institute of Allergy and Infectious Diseases, National Institutes of Health, Bethesda, MD 20892, United States; 3 Department of Biology, Loyola University Chicago, Chicago, IL 60660, United States; 4 These authors contributed equally to this work.

## Abstract

Control of parasite transmission is critical for the eradication of malaria. However, most antimalarial drugs are not active against *P. falciparum* gametocytes, responsible for the spread of malaria. Consequently, patients can remain infectious for weeks after the clearance of asexual parasites and clinical symptoms. Here we report the identification of 27 potent gametocytocidal compounds (IC_50_ < 1 μM) from screening 5,215 known drugs and compounds. All these compounds were active against three strains of gametocytes with different drug sensitivities and geographical origins, 3D7, HB3 and Dd2. Cheminformatic analysis revealed chemical signatures for *P. falciparum* sexual and asexual stages indicative of druggability and suggesting potential targets. Torin 2, a top lead compound (IC_50_ = 8 nM against gametocytes *in vitro*), completely blocked oocyst formation in a mouse model of transmission. These results provide critical new leads and potential targets to expand the repertoire of malaria transmission-blocking reagents.

Malaria cases and deaths have dropped 50% in 29 countries since 2000 due to the combined effects of long-lasting insecticidal bed nets, indoor residual spraying, and artemisinin-based combination therapies (ACTs)^1^. This success has raised hopes for malaria eradication and consequently stimulated interest in developing new reagents that block gametocyte transmission, such as novel and safe gametocytocidal drugs^2^. Previous drug development efforts have focused primarily on the asexual parasites that cause symptoms but not malaria transmission. To be transmitted from person to person via mosquitoes, the parasites must switch from asexual to sexual development and produce male and female gametocytes. Once gametocytes are taken up in a blood meal by a mosquito, fertilization is stimulated and the resulting zygote differentiates into a motile ookinete that migrates across the midgut epithelium and forms an oocyst. Over the course of the next 2 weeks, tens of thousands infectious sporozoites are generated and sequestered in the mosquito salivary glands until released into a vertebrate host for transmission during the next blood meal.

Sexual stage *P. falciparum* gametocytes have a lifespan of over 3 weeks and are not cleared effectively by current antimalarial agents, except primaquine (PQ)^3^,^4^, which is not widely used because it causes hemolytic anemia in patients with glucose-6-phosphate dehydrogenase deficiency^5^. Consequently, treatment with current antimalarial drugs often results in asymptomatic carriers who remain infectious for weeks after the clearance of asexual parasites. Despite the risks of PQ, its efficacy with artemisinin combination therapy (ACT) in reducing malaria transmission in the PQ-tolerant patients was recently demonstrated in test regions^6^. Other than PQ, the only other anti-gametocytocidal candidate being tested is methylene blue^7^. Thus, a new generation of antimalarial agents with potent activities against both sexual and asexual parasites is urgently needed for better therapeutic effect and eradication of malarial infection globally.

Due to the limited yield of gametocytes prepared from *in vitro* culture and assay sensitivity, high throughput gametocyte viability assays have only recently been developed^2^,^8^,^9^. We have screened 5,215 known compounds using the alamarBlue gametocyte viability assay and identified 27 novel gametocytocidal compounds. Because most of these compounds are approved drugs, a cheminformatic analysis of the screening data generated a profile of gametocytocidal compounds that were compared with those active against asexual parasites. These chemical signatures of known drugs suggest stage specific pathways as well as potential drug targets for both sexual (gametocytes) and asexual stages of the parasites including heat shock protein 90 (HSP90), aurora kinase (ARK1) and phosphatidylinositol 3-kinase (PI3K). A top lead compound, Torin 2, was confirmed with potent activities against both gametocytes and asexual parasites. Potential protein targets for this compound were also identified using affinity precipitation and drug affinity responsive target stability (DARTS)^10^. Furthermore, oocyst formation in mosquitoes was completely blocked by Torin 2 in a mouse model of transmission. Therefore, the identified lead gametocytocidal compounds as well as potential new drug targets and pathways essential for gametocyte development provide new directions for the design of the next generation antimalarial agents.

## Results

### Identification of 27 gametocytocidal compounds

**Figure 1:**
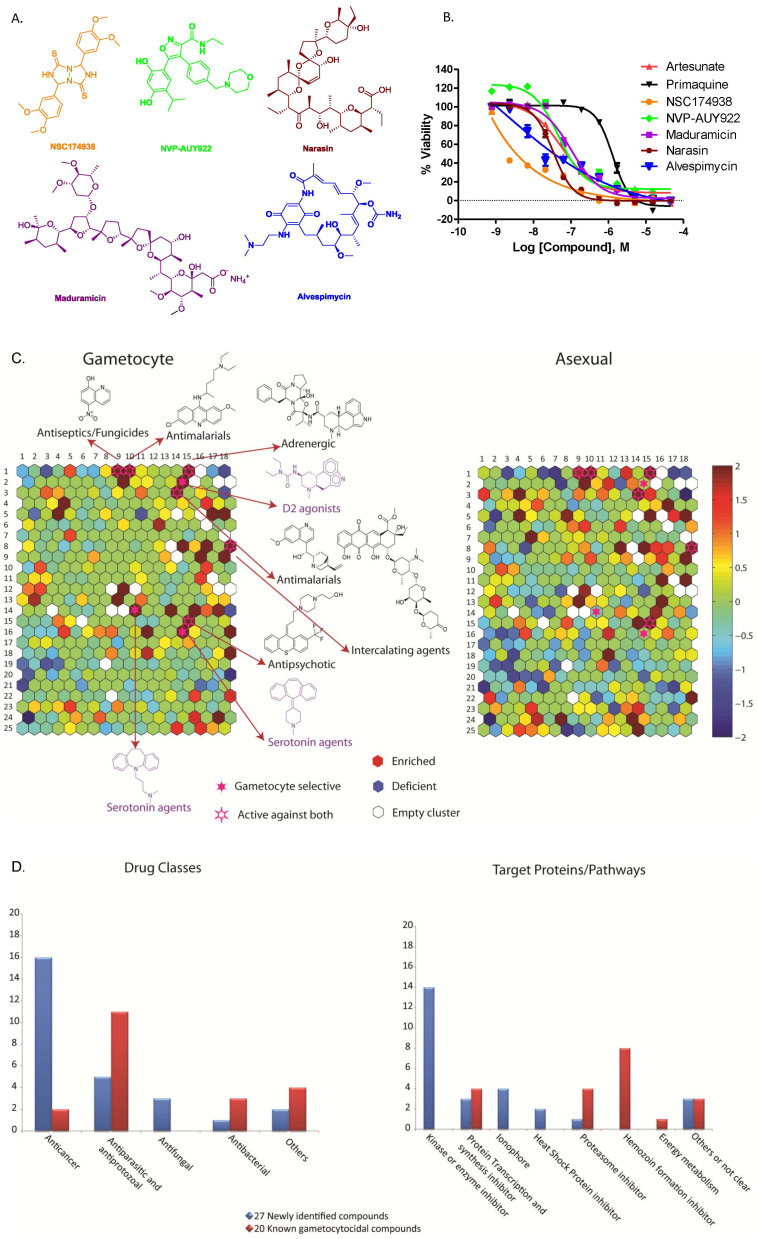
Cheminformatic summary of drug repurposing screen against *P. falciparum* 3D7 gametocytes. (A) Structures of top representative gametocytocidal compounds. (B) Concentration–response curves of selected lead compounds (NSC174938, NVP-AUY922, maduramicin, narasin, alvespimycin, primaquine and artesunate) determined in the gametocyte viability assay. (C) Structure clustering of compound activity across the compounds screened. In the heat maps, each hexagon represents a cluster of compounds with structural similarity. Red colored clusters represent structures enriched in compounds active against the parasites as measured by a Fisher's exact test. Blue colored clusters represent structures with minimal active compounds. Coloring is scaled by the negative log10 of the P-values. Darker in red or blue color indicates a higher level of enrichment or absence of active compounds in each structure cluster. Compound structures show the examples of known drug groups active against both gametocytes and asexual parasites (red hexagons in both heat maps) or selectively active against gametocytes over asexual parasites (red hexagons in the gametocyte map and greenish or blue in the asexual map with structures and annotations highlighted in purple). (D) Distribution of known drug indications and targets/pathways of 27 newly identified gametocytocidal compounds compared to 20 previously reported gametocytocidal compounds. Left panel: number of active compounds in each drug class. If a compound has more than one indication, it is counted once by the following order: antiparasitic and antiprotozoal, antifungal, antibacterial, anticancer or others. Right panel: number of active compounds in each known drug targets/pathways.

*P. falciparum* strain 3D7 gametocytes were screened against 5,215 compounds at four concentrations ranging from 0.37 to 46 μM using an alamarBlue viability assay^9^,^11^. These compounds include 4,265 approved human or animal drugs^12^, 400 from the Malaria Box library that are active against *P. falciparum* strain 3D7 asexual parasites *in vitro*^13^, and 550 from an internal collection of kinase inhibitors^14^. A total of 27 novel active gametocytocidal compounds were identified and confirmed with IC_50_ values ≤1 μM against gametocytes. Among these confirmed compounds, 21 had more than 10-fold selectivity against gametocytes over the mammalian cell line HepG2 (Table 1 and SI Table 1). Gametocytocidal activities of these 27 compounds have not been previously reported elsewhere. NSC174938, Torin 2, NVP-AUY922, maduramicin, and narasin were the most potent compounds against gametocytes with IC_50_ values ranging from 3 to 50 nM (Fig. 1A, 1B, and Table 1). Two compounds in the malaria box reduced gametocyte viability by >75% and had an IC_50_ of <1 μM, MMV019406 and MMV666125 (SI Table 2). Additionally, PQ and 7 other compounds with known gametocytocidal activity were present in the compound collection and were all identified in the screen (Table 1), validating the effectiveness of this screening method. The IC_50_s of the endoperoxides were similar to those previously reported for gametocyte stages IV-V^15^, but lower than the IC_50_s recently reported using an ATPase assay^16^. The reason for this difference is unknown, although the length of drug exposure and gametocyte purification methods varied among these assays.

### Cheminformatic analysis of gametocytocidal activity compared to activity against asexual parasites

In addition to the 27 potent compounds analyzed above, many others among the 5,215 compounds screened also exhibited gametocytocidal activity. Most of the compounds screened in this experiment had been previously profiled against the asexual stages of *P. falciparum* strain 3D7 and its clinical variants^17^,^18^. These two previous studies demonstrate the utility of profiling chemical genomic signatures of asexual parasites by screening the approved drug collection. To identify structural motifs that are selectively active against gametocytes over asexual parasites, we clustered these compounds based on their structural similarity (2,048-bit Daylight fingerprints; Daylight Chemical Information Systems, Inc., Laguna Niguel, CA) using the self-organizing map (SOM) algorithm^19^ (Fig. 1C). Each hexagon in the SOMs represents a cluster of structurally similar compounds, with neighboring hexagons containing more similar structures than distal hexagons and is designated by their row and column number. This analysis identified a number of compound classes that are active against both gametocytes and asexual parasites (Fig. 1C, hexagons colored red in both the gametocyte and the asexual SOMs), including DNA intercalating agents (8.18), certain classes of antipsychotic drugs (15.15) and adrenergic agents (1.15), as well as the groups containing the antimalarials, primaquine (1.10) and mefloquine (3.14) and classes of antiseptics and fungicides (1.9). We also found structural motifs that appear to be specific against gametocytes (SI Table 3 and Fig. 1C, hexagons colored red in the gametocyte SOM but greenish or blue in the asexual SOM), exemplified by a class of D2 agonists (2.14) and certain classes of serotonin agents (14.10 and 16.14). The analysis also confirmed the specificity of dihydrofolate reductase (DHFR) inhibitors against asexual stages of parasites^20^. Together, the results indicate the druggability of *P. falciparum* gametocytes by chemical motifs that exist in approved drugs as well as selectivity of these compounds against the gametocytes.

### New targets for gametocytocidal drug development

Many active compounds identified by screening a known drug library cannot be directly used in patients because effective compound concentrations in plasma or tissue for the new indication cannot be achieved. However, compounds with known mechanism of action provide valuable information for identification of their targets. These compounds can be optimized, or new compounds can be developed against the newly identified drug targets. In this case, the selective compounds may be developed based on the significant differences between malarial and human proteins. Because most screened compounds are known drugs or pharmacologically active compounds, we further analyzed the parasite stage specific compounds to identify potential new targets for gametocytocidal drug development and pathways potentially required for gametocyte development. Compound activities against *P. falciparum* gametocytes were also compared with those obtained in previous screens against asexual parasites. Based on compound selectivity, three groups of compounds were identified: (1) those selective against gametocytes, (2) those selective against asexual parasites, and (3) those active against both stages of the parasites (SI Table 4). Each group was tested for enrichment with known targets and/or therapeutic indications (significantly enriched, Fisher's exact test; p < 0.05). We found that drugs acting on cholinergic receptors (muscarinic 1 and 2), 5-Hydroxytryptamine (serotonin) receptor 2A and solute carrier family 6 are gametocyte-selective. Interestingly, no close homologues for these proteins have been found in *P. falciparum*. However, a tricyclic structure is a common theme present in these active compounds, indicating that this structure may bind well to a putative gametocyte-specific protein. In contrast, drugs acting on DHFR are selective for asexual parasites, which is consistent with a previous report^21^. And compounds active against topoisomerases and potassium voltage-gated channels are effective on both gametocytes and asexual parasites (SI Table 4).

Additionally, drug indication analysis of the top 27 potent gametocytocidal compounds revealed that 59% are known anticancer agents, while only 19% are previously established antiparasitic/antiprotozoal agents. The remaining 22% are categorized as antibacterials, antifungals or others (Fig. 1D). Furthermore, analysis of compounds with known drug targets revealed that 52% of newly identified target proteins are kinases/other enzymes including ARK1^22^, and histone deacetylase (HDAC)^23^, 11% are proteins involved in protein transcription/synthesis, 7% are heat shock proteins, 4% are proteasome components and 11% have other functions. The remaining 15% of the compounds are classified as ionophores (Fig. 1D). However, annotated *P. falciparum* homologues could only be found for seven of the target enzymes predicted by prior analysis of the approved drugs on mammalian cells (SI Table 5). By comparison, 55% of the 20 previously identified gametocytocidal compounds (Table 1 and SI Table 6), except PQ^24^, are more potent against asexual parasites than gametocytes, which accounts for the ineffectiveness in blocking malaria transmission by routine antimalarial treatment^25^,^26^. Additionally, 40% of the 20 previously identified gametocytocidal compounds including the four artemisinin derivatives tested have been reported to affect hemozoin formation, 20% target the proteasome, 20% affect DNA transcription, 5% are related to energy metabolism, and 15% have other functions. Together, we have identified several groups of new gametocyte targets, providing alternative approaches for future antimalarial drug development.

### Profiles of gametocytocidal compounds against drug resistant strains

**Figure 2:**
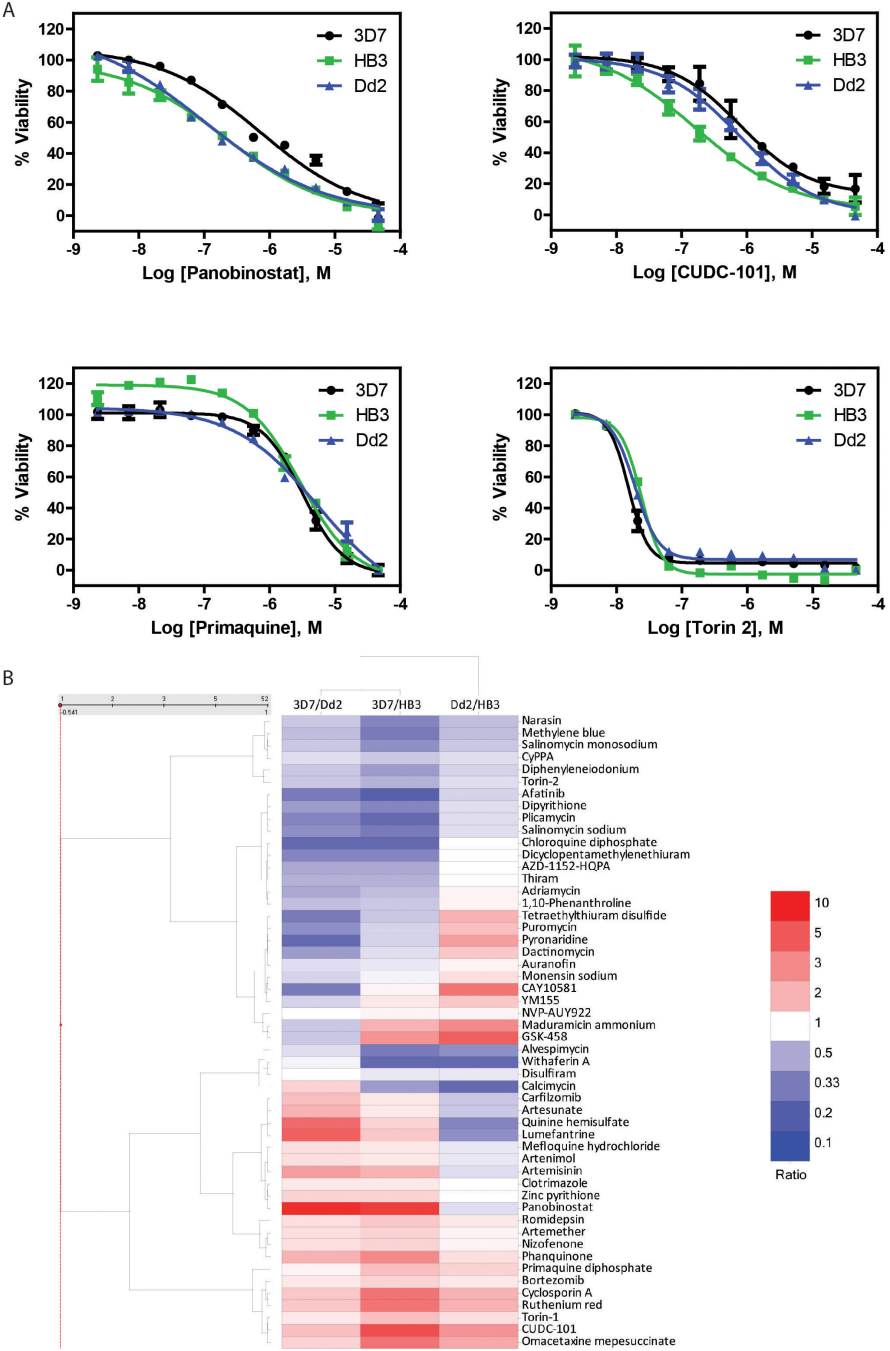
Profiling of gametocytocidal compounds against *P. falciparum* gametocytes of drug sensitive strain 3D7 and drug resistant strains HB3 and Dd2. Asexual Dd2 parasites are resistant to chloroquine, mefloquine, and pyrimethamine while HB3 is resistant to pyrimethamine but not chloroquine or mefloquine. (A) Concentration-response curves of two representative strain selective compounds panobinostat and CUDC-101 (with potencies greater than 5-fold to HB3/Dd2 strains) in comparison with strain nonselective compounds primaquine and Torin 2. (B) Comparison of compound potencies across three strains. For example, a compound colored in white in 3D7/Dd2 group indicates equal potencies in both strains, whereas the one in dark red indicates that the compound is more potent against Dd2 and the one in dark blue means that the compound is more potent against 3D7. Compounds in the heat map are hierarchically clustered by their IC_50_ ratio profile across the three strains.

Drug resistance is also a critical challenge for malaria treatment and eradication that has not been examined in gametocytes, though it has been extensively studied for the asexual parasites^27^,^28^. To evaluate whether existing antimalarial agents and newly identified gametocytocidal compounds are effective against well characterized drug resistant strains, we determined the gametocytocidal activities of 52 selected compounds, including 27 newly identified compounds and 25 known antimalarial agents, against gametocytes of *P. falciparum* strains Dd2 and HB3 in the alamarBlue viability assay. In contrast to 3D7, asexual Dd2 parasites are resistant to chloroquine, mefloquine and pyrimethamine while asexual HB3 parasites are resistant to pyrimethamine but not chloroquine or mefloquine^29^. Most of 52 compounds showed 5-fold or less differences in potency between these two parasite strains compared to the drug sensitive stain 3D7 (Fig. 2A, 2B and SI Table 7). Compared to the drug sensitive stain 3D7, chloroquine's potency against Dd2 gametocytes was reduced 3.7-fold while it was 10-fold less potent against Dd2 asexual parasites^29^. Methylene blue was moderately more active against 3D7 gametocytes (IC_50_ = 0.307 μM) than those of HB3 (IC_50_ = 0.935 μM) and Dd2 (IC_50_ = 0.526 μM) (SI Table 7). PQ showed similar potencies against gametocytes from these three strains with IC_50_ values of 1.26, 0.68, and 1.08 μM against 3D7, HB3, and Dd2, respectively (Fig. 2A).

Interestingly, several of these newly identified gametocytocidal compounds exhibited similar or higher activities in these two asexual drug resistant strains compared to the drug sensitive 3D7 strain. For example, CUDC-101, a multi-target anticancer drug candidate^30^, was 5.5-fold more potent against HB3 (IC_50_ = 0.152 μM) compared to 3D7 (IC_50_ = 0.833 μM) (Fig. 2A). Additionally, panobinostat, a histone deacetylase inhibitor, was also 6.3 to 7.9 times more potent against Dd2 (IC_50_ = 0.148 μM) and HB3 (IC_50_ = 0.118 μM) compared to 3D7 (IC_50_ = 0.935 μM) (Fig. 2A). These results suggest that these newly identified gametocytocidal compounds could also be effective against a range of asexual drug resistant isolates.

### Activities of Torin 2 against gametocytes and asexual parasites *in vitro*

**Figure 3:**
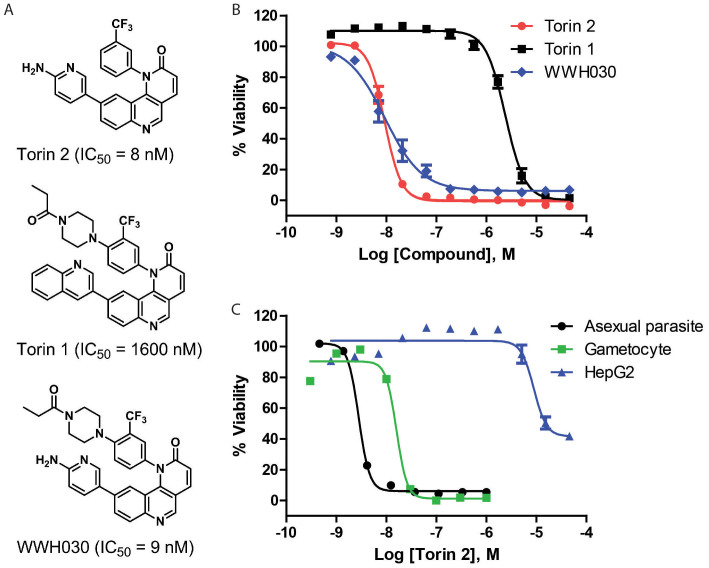
Structures and activities of Torin 2 and its analogs. (A) Chemical structures of Torin 2, Torin 1 and WWH030. (B) Concentration–response curves of Torin 2, Torin 1 and WWH030 against gametocytes measured in the high throughout viability assay. Torin 1 was much weaker in potency compared to Torin 2. (C) Concentration-response curves of Torin 2 determined in the optic microscopic gametocyte assay, in the asexual parasite SYBR green assays and in the mammalian HepG2 cell cytotoxicity assay. Torin 2 exhibited a great selectivity to malaria parasites over the mammalian cells.

Torin 2, a known mTOR inhibitor^31^, was one of the most potent new gametocytocidal compounds (IC_50_ = 8 nM, Table 1). In contrast, its structural analog, Torin 1, was 200-fold less potent (IC_50_ = 1.6 μM, Fig. 3B), regardless of their similar potencies on mTOR (IC_50_ values of 5.4 and 2.1 nM, respectively)^32^. The difference in gametocytocidal activity between the two compounds was confirmed using the traditional gametocyte viability assay, optical microscopy of Giemsa stained smears (SI Fig. 2A). Both Torin 1 and 2 share the same core structure, benzo[h][1,6]naphthyridin-2(1H)-one, but the 2-aminopyridine in Torin 2 is replaced with a quinolone in Torin 1 (Fig. 3A). The 200-fold difference in potencies against *P. falciparum* gametocytes suggests that Torin 2 and Torin 1 may act on a target or targets other than mTOR, consistent with the lack of mTOR homolog in *P. falciparum*^33^.

To further characterize the utility of Torin 2 as an antimalarial drug, its *in vitro* activity against asexual blood stage parasites and HepG2 cells was also evaluated. Torin 2 exhibited an IC_50_ of 2.75 nM (Fig. 3C) against asexual parasites, while Torin 1 had an IC_50_ of 215 nM (SI Fig. 2B), making Torin 1 78-times less potent than Torin 2. Notably, both compounds were more potent against the asexual parasites than gametocytes. Additionally, Torin-2 toxicity in mammalian cells was assessed using HepG2 cells (Fig. 3C and SI Table 1). Torin 2 exhibited only partial cytotoxicity at the highest tested concentration (46 μM), indicative of greater than 1,000-fold selectivity against the parasites over the mammalian cells (SI Table 1). Taken together, the results demonstrate the similar low nanomolar potencies of Torin 2 against both sexual and asexual stages of *P. falciparum*, as well as its high selectivity against *P. falciparum* parasites over mammalian cells.

### Efficacy of Torin 2 on gametocyte transmission from vertebrate host to mosquitoes in a mouse model

**Figure 4:**
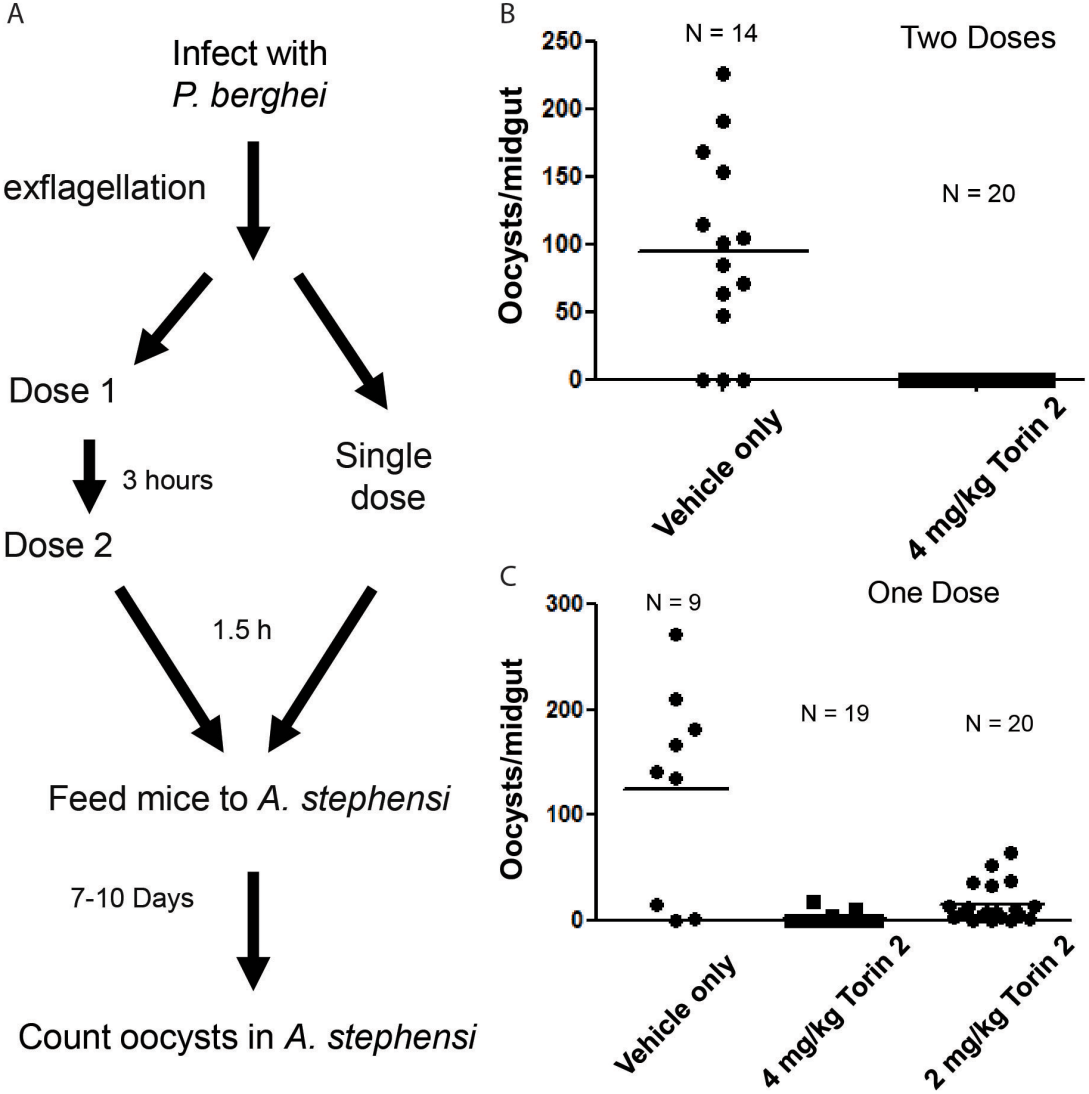
*In vivo* efficacy of Torin 2 in a mouse model of transmission. (A) Schematic process of mouse–mosquito transmission using *P. berghei* and *A. stephensi*. Mice infected with *P. berghei* were treated with drug or vehicle alone. 1.5 hr after drug treatment, *A. stephensi* was allowed to feed on the infected mice to test parasite transmission. *A. stephensi* were maintained for 10 days to allow oocyst development, and the numbers of oocysts per mosquito midgut were recorded. (B) Two doses of 4 mg/kg Torin 2 given 3 hrs apart completely prevented oocyst formation in mosquitoes that fed on the infected mice 1.5 hrs post drug treatment. A representative experiment is shown (p < 0.001, Student's T-test). (C) A single dose of 2 mg/kg Torin 2 significantly reduced oocyst production in contrast to nearly complete elimination of oocyst production by a single dose of 4 mg/kg Torin 2 (p < 0.001, one way ANOVA with Tukey's post-hoc test).

The transmission of *Plasmodium berghei* ANKA (*Pb*) from infected mice to *Anopheles stephensi* mosquitoes was examined to investigate the *in vivo* efficacy of Torin 2. The experiment was designed to accommodate developmental differences between *P. berghei* and *P. falciparum*. In contrast to *P. falciparum*, which requires 48 hrs to complete its asexual life cycle and 10–12 days to complete sexual differentiation, *P. berghei* completes asexual replication and sexual differentiation cycles in 24 hrs. *Pb*-infected mice were assessed for exflagellation to monitor the presence of *P. berghei* gametocytes, and drug or vehicle control were administered when >1 exflagellation center per two 400× field were observed. Some mice were given two doses of drug three hrs apart to increase exposure time, but on a short enough time scale to prevent a concomitant decrease in asexual parasite from affecting the next cycle of gametocytes. This dosing schedule was based on the transmission experiments done with primaquine^34^. The mosquito feed assay was performed 1.5 hrs after the final drug treatment as described in the Methods (Fig. 4A). Exflagellation was also monitored just prior to mosquito feed, and no difference was observed between the drug- and vehicle-treated mice (data not shown). In contrast, oocyst production in the fed mosquitoes, which was used as an indicator of malaria transmission, was completely blocked by treatment with two 4 mg/kg doses of Torin 2 in 3 independent experiments while 73% of the mosquitoes fed on control mice had >45 oocysts/midgut (Fig. 4B, p < 0.001, Students T-test). To further evaluate the dose dependence, we tested single 2 or 4 mg/kg doses of Torin 2 in the same mouse model (Fig. 4C). A single dose of 2 mg/kg of Torin 2 significantly reduced oocyst production, while a single 4 mg/kg dose almost completely eliminated it. These results clearly demonstrate the ability of Torin 2 to prevent oocyst formation in mosquitoes in a dose dependent manner.

### Identification of potential molecular targets of Torin 2

**Figure 5:**
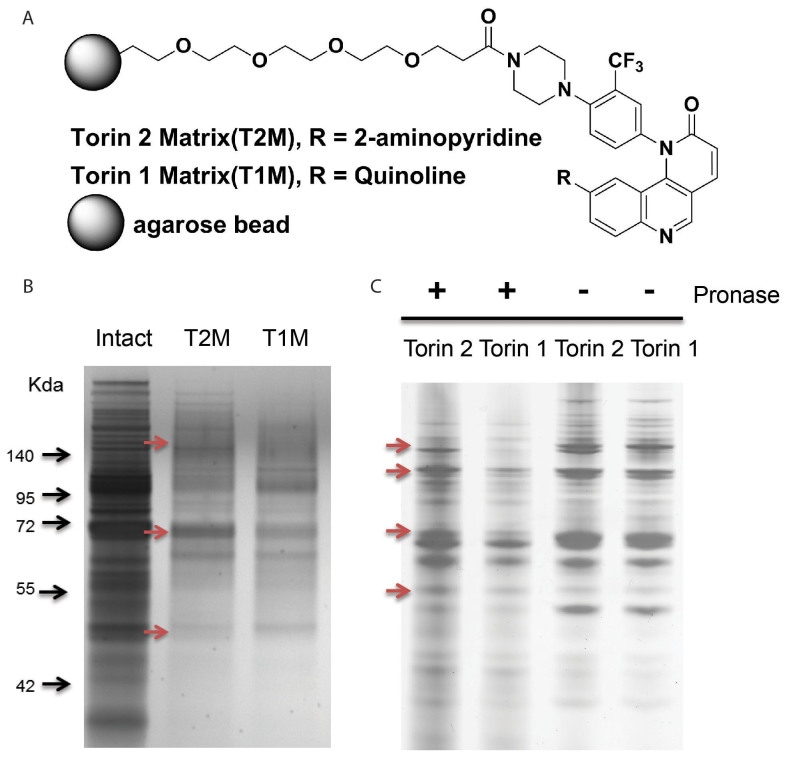
Target identification for potential Torin 2 interacting proteins by affinity precipitation and drug affinity responsive target stability (DARTS). (A) Chemical structures of Torin 2 matrix (T2M) and Torin 1 matrix (T1M) for the affinity precipitation (pull-down) experiment. Torin 1 matrix probe was used as a negative control. (B) Protein bands identified by the affinity precipitation experiment using T2M in comparison with T1M negative control. *P. falciparum* 3D7 gametocyte lysates were incubated with the affinity probes at 4°C for 2 hrs. Bound proteins were eluted, resolved on a 10% Bis-Tris gel, and visualized with silver staining. The arrows indicated protein bands (selective to T2M probe) were processed for mass spectrum analysis. (C) Protein bands identified by the DARTS experiment. *P. falciparum* 3D7 gametocyte lysates were treated with either Torin 2 (lane 1 and 3) or Torin 1 (lane 2 and 4) at a concentration of 600 nM for 1 hr. Each sample was split into two aliquots for proteolysis with (lane 1 and 2) or without (lane 3 and 4) pronase. The partially digested proteins were separated by 10% Bis-Tri gel and visualized with silver staining. The Torin 2 specific protein bands indicated by arrows were further processed for mass spectrum analysis.

The lack of an mTOR homologue in *P. falciparum*^33^ and the significant difference in the potencies of Torin 1 and Torin 2 against the parasites suggest the presence of distinct targets in the parasites. We hypothesized that Torin 2 selectively interacts with an unknown *P. falciparum* protein (or proteins) that has a weaker binding affinity to Torin 1. To develop a probe for an affinity based pull-down experiment, a Torin derivative, WWH030, was synthesized, and the importance of the ortho-peperzine-amide on the (trifluoromethyl)-benzene of Torin 2 for its gametocytocidal activity was determined (Fig. 3A). The new derivative had an IC_50_ of 9 nM, similar to that of Torin 2 in the gametocyte assay (Fig. 3 B). This result indicates that the ortho-peperzine-amide group on Torin 2 can be modified without a significant effect on its gametocytocidal activity. Therefore, T2M was synthesized as an affinity resin for the pull-down experiment for identification of Torin 2 interacting proteins in *P. falciparum* gametocyte lysates (Fig. 5A). A negative control resin, T1M, was similarly synthesized with a close analog of Torin 1 (Fig. 5A).

The proteins precipitated from gametocyte lysate by T2M but not T1M were identified by mass spectrometric analysis (Fig. 5B)^35^. The proteomics data revealed a total of 31 proteins selectively enriched by T2M (SI Table 8). In parallel to the probe-protein precipitation experiment, we also carried out a DARTS experiment^10^ to identify Torin 2 binding proteins by limited protease digestion of Torin 2-treated gametocyte lysates. Following treatment with either Torin 2 or the negative control Torin 1, gametocyte lysates were partially digested with pronase and size fractionated by SDS-PAGE (Fig. 5C). Four significant protein bands were enriched in the Torin 2-treated sample compared to the Torin 1-treated sample and analyzed by mass spectrometry. After comparing the results from the affinity precipitation experiment, we found that phosphoribosylpyrophosphate synthetase (PF3D7_1325100, ribose-phosphate diphosphokinase), aspartate carbamoyltransferase (PF3D7_1344800, ATCase), and a putative transporter (PF3D7_0914700) were identified by both experiments (Table 2). Thus, these three gametocyte proteins are potential drug targets for Torin 2 and will need to be further confirmed by enzyme assays and binding assays using recombinant *P. falciaprum* proteins.

## Discussion

Taking advantage of the miniaturized gametocyte viability assay, we have screened 5,215 approved drugs and known compounds to profile the gametocytocidal activity of these compounds. Among the 27 potent gametocytocidal compounds identified, 16 are completely new to the malaria research community and the other 11 have known activity against asexual parasites (Table 1). The chemical informatics analysis of these compounds in combination with their known mechanisms of action have revealed a set of target proteins such as HSP90, HDAC, ARK1, PI3K, S-adenosylhomocysteine hydrolase (SAHH), and thioredoxin reductase that are crucial for the growth and survival of malaria. Thus, these newly identified protein targets provide a new direction for the study of essential signaling pathways in the parasites and the development of antimalarial agents against gametocytes.

Structurally similar compounds in the same chemical cluster often share a similar mode of action^36^. This approach is especially useful for phenotypic drug screens where specific target information is unavailable. Our cheminformatic analysis of compound activities obtained in this study and those previously reported for asexual parasites revealed both shared and distinct chemical signatures against gametocytes and asexual parasites (Fig. 1C). The analysis revealed several core templates (e.g. quinine and quinacrine types of antimalarial agents, anthracyclines, and dihydroergotamine type of adrenergic agents) that are active on both stages of the parasites. Dibenzazepine class of serotonin agents are a structure class that is specifically active in gametocyte stage, indicative of potential targets selectively expressed in gametocytes. The activities of antimalarial quinine analogs and anthracycline derivatives against both asexual parasites and gametocytes have been reported previously^37^,^38^, validating the utility of this analysis method. Dihydroergotamine types of adrenergic agents are a new cluster of compounds active against both asexual parasites and gametocytes, whose activities were previously shown only against *P. falciparum* asexual parasites^18^. These observations indicate that the structurally similar compounds in the same clusters may share common modes of actions.

Kinases and other enzymes make up the largest percentage of potential protein targets identified from this study for gametocytocidal drug development. 48% of the active compounds in the previous screen against *P. falciparum* asexual parasites targeted kinases^40^, reflecting the abundance of these compounds in the library. Less than 100 kinases have been identified in *P. falciparum*, compared to over 450 kinases in human^39^. Our new data in combination with the data from previous reports for asexual parasites^40^,^41^ indicate that malaria kinases are a group of potential targets for development of new antimalarial agents that control both sexual and asexual parasites.

Additionally, two anticancer agents (NVP-AUY922 and alvespimycin), two ionophores (maduramicin and narasin) and the mTOR inhibitor Torin 2 exhibited potent activities against gametocytes of all three tested *P. falciparum* strains including two previously reported asexual drug resistant ones. NVP-AUY922 and alvespimycin are both potent inhibitors of HSP90^42^,^43^. Currently NVP-AUY922 is being used as an experimental drug in late human clinical trials for the treatment of metastatic non-small cell lung cancer^44^ and alvespimycin has been tested in patients with advanced solid tumors^45^. Alvespimysin is a derivative of geldanamycin, a natural product that was known to kill asexual parasites, but its effect on malaria transmission was not tested^46^. Heat shock proteins have been extensively studied in *P. falciparum* and are predicted to be antimalarial targets, but their effects on gametocytes have not been reported before^46^,^47^,^48^. There are four *P. falciparum* HSP90 homologs including Pf3D7_708400, Pf3D7_1443900, Pf3D7_118200, and HSP90b1 P3D7_ 1222300 which share 33 to 68% homology with human HSP90s^49^. Pf3D7_708400 and Pf3D7_118200 are expressed throughout parasite development including stage V gametocytes, indicative of potential targets for antimalarial agents against multi-stage parasites.

Torin 2 is another strong candidate for further development. It effectively kills both asexual parasites and gametocytes of all three *P. falciparum* strains tested with similar potencies including the asexual drug resistant strains, HB3 and Dd2^29^. It also completely blocked *Pb* oocyst production in the mouse-mosquito transmission model and showed more than 1,000-fold selectivity for the parasites over mammalian cells. This compound has been reported as a potent second generation ATP-competitive inhibitor of mTOR^32^ with good oral bioavailability (51%). In mice, its concentrations in plasma, liver and lung maintained strong mTOR inhibition for 6 hours after a single dose of 20 mg/kg^32^. The antitumor activity of Torin 2 was observed in KRAS-driven lung tumors in combination with mitogen-activated protein/extracellular signal–regulated kinase (MEK) inhibitor AZD6244^31^. The mammalian lipid kinase profiling of Torin 2 has been extensively studied and revealed that in addition to mTOR, phosphatidylinositol-3 kinase–like kinase (PIKK) family kinases are major Torin 2 interacting proteins^31^,^32^. During the preparation of this manuscript, another group reported that Torin 2 inhibited *P. falciparum* development in the liver stage (IC_50_ = 1.1 nM), as well as the asexual blood stage (IC_50_ = 1.4 nM) and the early stages (I and II) of *P. falciparum* gametocytes (IC_50_ = 6.6 nM)^51^. In this study, we found that Torin 2 potently killed the gametocyte stages III-V with nanomolar activity and completely blocked gametocyte transmission to mosquitoes *in vivo* using the mouse model. This work complements the recent report on the effects of Torin 2 on the liver and asexual stages and early gametocytes^50^. Together, the data demonstrate that Torin 2 is effective against sexual and asexual blood stages as well as liver stages of malaria parasites with low nanomolar potency and blocks transmission to mosquitoes at a tolerable dose and thus is an ideal lead compound for the next generation antimalarial agent.

Though Torin 2 was able to prevent oocyst formation in mosquitoes that fed on infected mice, the precise stage of the life cycle affected is unknown. In the *P. berghei* mouse model, parasite exflagellation in the drug-treated animals was equivalent to that of the vehicle-treated controls. These data indicate that the male *P. berghei* gametocytes are still viable 1.5 hrs after drug treatment. It is possible that some amount of Torin 2 carried over into the mosquitoes and affected one of the stages between the gametocyte and the oocyst: gamete, zygote or ookinete. Alternatively, Torin 2 could be affecting a parasite pathway that allows exflagellation, but blocks subsequent steps that are necessary for productive mosquito infection. In either case, Torin 2 remained active *in vivo* and effectively blocked oocyst production. Future studies will examine which of these stages are affected by Torin 2 using *P. berghei* ookinete cultures.

An mTOR homolog has not been identified in *P. falciparum*, and the homologies between parasite PIKK and mammalian PIKK are not striking. There is only 22% identity in the 148 amino acid catalytic domain of PfPI3K and HsPI3K^33^. To identify the target of Torin 2 in *P. falciparum*, we have employed two independent approaches, Torin 2 affinity precipitation and DARTS. Phosphoribosylpyrophosphate synthetase (RPPK), aspartate carbamoyltransferase (ATCase), and a putative transporter PF3D7_0914700 were found in both experiments as the potential targets of Torin 2. RPPK is a kinase that catalyzes the synthesis of PRPP and adenosine monophosphate (AMP) from ribose 5-phosphate and adenosine 5′-triphosphate (ATP)^51^. Although its activity has not been directly tested in *P. falciparum*, this enzyme links carbon and nitrogen metabolism and is essential for the salvage and synthesis of purines as well as the synthesis of pyrimidines and pyridines in many species from yeast to humans^52^. It also plays a role in histidine and tryptophan metabolism in bacteria and fungi^53^. In contrast, ATCase catalyzes the first step in the pyrimidine biosynthetic pathway^54^. The participation of these two enzymes in pyrimidine synthesis suggests that the very early steps in this pathway are important for gametocyte growth and survival, which have not been previously recognized as potential targets for gametocytocidal drug development. The third potential target is predicted to be a transporter, which may involve the transfer of charged molecules across cell membrane lipid bilayers. These results open a door to further study gametocyte targets, allowing the understanding the mechanism of action for Torin 2 and accelerating the further development of this compound. Similarly, serotonin antagonists were found as potent gametocytocidal compounds although there is no *P. falciparum* homolog of serotonin receptors, indicative of the existence of a potential gametocyte specific target.

In summary, the cheminformatic analysis of chemical signatures of the active compounds identified from this study against the sexual stages (gametocytes) and asexual blood stages of *P. falciparum* (previously reported) have revealed new chemical signatures and protein targets for future antimalarial drug development. Among 27 newly identified compounds, Torin 2 possesses nanomolar potencies against both asexual and sexual *P. falciparum* parasites with the ability to completely block gametocyte transmission from host to mosquitoes. In addition, RPPK, ATCase, and transporter (PF3D7_0914700) were identified as potential targets of Torin 2. Therefore, this study provides critical new leads and novel targets to accelerate gametocytocidal drug development. We also think that this approach can be extended to other pathogens including bacteria, fungi, protozoa and parasites for rapid identification of new drugs and drug targets.

## Methods

### Cell culture

Asexual parasites of *P. falciparum* strain 3D7 were cultured as described previously^55^. Briefly, gametocyte cultures were set up at 0.1% parasitemia and on days 9–10 treated with 50 mM N-acetylglucosamine (NAG) to block further asexual growth. Stage III–V gametocytes were isolated by Percoll density gradient centrifugation on day 12 and returned to culture for 24 hrs before being used in the assay^9^. At the time of the assay 73% of the gametocytes were stage IV or V. Gametocytes of HB3 and Dd2 strains were produced and then set up for assay in a similar process. HepG2 cells (ATCC, cat. no. 77400) were cultured in 175-cm^2^ tissue culture flasks with 30 ml growth medium at 37°C in a 5% CO_2_ & 5% O_2_ humidified atmosphere. Growth medium was made with Dulbecco's Modified Eagle Medium with 10% fetal bovine serum (FBS). Growth medium was replaced every other day and cells were passed at 75% confluence.

### Compound library and gametocyte assay screen

The National Institutes of Health (NIH) Chemical Genomics Center Pharmaceutical Collection (NPC) was constructed in-house through a combination source of traditional chemical suppliers, specialty collections, pharmacies and custom synthesis^12^. Briefly, the NPC library comprises 4,265 small-molecule compounds, 49% of which are drugs approved for human or animal use by the US Food and Drug Administration (FDA), 23% are drugs approved in Canada/UK/EU/Japan, and the remaining 28% are compounds that have entered clinical trials or are research compounds commonly used in biomedical research. The Malaria Box contains 400 drugs or tool compounds that have confirmed activity on blood-staged *P. falciparum* and assessed cytotoxicity against mammalian cells^40^,^56^. The MIPE library is an internal collection of 550 kinase inhibitors, which contain approved drugs and compounds in clinical and preclinical stages^14^. Compounds from all libraries were obtained as powder samples and dissolved in DMSO as 10 mM stock solutions, except several hundred from the NPC library that were prepared as 4.47 mM stock solutions due to solubility limitations.

Compound screening experiments were performed as previously described^11^. Briefly, 2.5 μl/well incomplete medium was dispensed into each well of 1,536-well plates using the Multidrop Combi followed by 23 nl compound transferring using the NX-TR Pintool (WAKO Scientific Solutions, San Diego, CA). Then, 2.5 μl/well of gametocytes was dispensed with a seeding density of 20,000 cells/well using the Multidrop Combi. The assay plates were incubated for 72 hrs at 37°C with 5% CO_2_. After addition of 5 μl/well of 2× AlamarBlue dye (Life Technologies, cat. no. DAL1100), the plates were incubated for 24 hrs at 37°C with 5% CO_2_ and then were read in a fluorescence detection mode (Ex = 525 nm, Em = 598 nm) on a ViewLux plate reader (PerkinElmer).

### Small molecule pull-down

Affinity matrix: To make a bead-connected affinity probe of Torin 2, a tetraethylene glycol linker was attached to 1-(piperazin-1-yl)propan-1-one of HWW030 and then coupled to Affi-Gel 10 resin (Bio-Rad Laboratories, cat. no. 153-6046) under mild basic conditions to afford Torin 2 matrix (T2M). See detailed version in SI methods. Torin 1 was similarly immobilized to resin and used as a negative control (T1M). The resultant affinity probes were incubated with gametocyte lysates, the bound proteins were eluted from resin by boiling in SDS-PAGE sample loading buffer. The eluted fractions were separated by SDS-PAGE and visualized by silver staining. RBC infected with gametocytes (3D7 strain: Stages III-V) were washed 3 times with PBS and then lysed by 0.05% saponin treatment in PBS for 5 mins at room temperature. The prepared gametocytes were washed 3 times with PBS and frozen at minus 80°C. The affinity precipitation experiment was processed as previously described^35^. The frozen samples were lysed with homogenization buffer (60 mM glycerophosphate, 15 mM p-nitrophenyl phosphate, 25 mM MOPS (pH 7.2), 15 mM EGTA, 15 mM MgCl2, 1 mM DTT, protease inhibitors (Roche Diagnostics, cat. no. 11836170001), and 0.5% Nonidet P-40). Cell lysates were centrifuged at 16,000 × g for 20 mins at 4°C, and the supernatant was collected. Protein concentration in the supernatant was determined by using a BCA protein assay kit (Pierce Chemical, cat. no. 23225). The lysate (0.5 mg) was then added to the packed affinity matrix, and bead buffer (50 mM Tris HCl (pH 7.4), 5 mM NaF, 250 mM NaCl, 5 mM EDTA, 5 mM EGTA, protease inhibitors, and 0.1% Nonidet P-40) was added to a final volume of 1 ml. After rotating at 4°C for 2 hrs, the mixture was centrifuged at 16,000 × *g* for 2 mins at 4°C, and the supernatant was removed. The affinity matrix was then washed (six times) with cold bead buffer and eluted by boiling with SDS-PAGE sample loading buffer at 95°C for 5 mins. Supernatants were separated on a 10% Bis-Tris gel (Life Technologies) and visualized by silver staining using a Pierce Silver Stain Kit for Mass Spectrometry (Pierce Chemical).

### DARTS

The 3D7 gametocytes were lysed with M-PER (Peirce Chemical) supplemented with protease and phosphatase inhibitors as previously described^10^. After centrifugation at 16,000 × *g* for 20 mins, protein concentration in the supernatant was quantified and 2 μg/μl proteins were treated with 600 nM of Torin 2 or 600 nM of Torin 1 for 2 hrs at room temperature. The samples were treated with 46 μg/ml pronase (Sigma-Aldrich, cat. no. P6911) for 30 mins at room temperature. The digestion was stopped by adding the SDS-PAGE sample loading buffer and boiled at 70°C for 10 mins. The samples were separated on a 10% Bis-Tris gel and visualized by silver staining.

### Mouse malaria model

*Plasmodium berghei* ANKA (Pb) parasites were maintained by serial passage by intraperitoneal (i.p.) injection in outbred mice. All parasites used were serially passaged less than 5 times. Two days before feeding, female mice were infected i.p. with 200–400 μl whole blood from a Pb-infected mouse with >10% parasitemia, resulting in a high parasitemia in a short, predictable amount of time. On day 2 post infection, the mice were checked for exflagellation. If exflagellation was not observed, it was reassessed on day 3. All mice exhibited exflagellation on either day 2 or day 3. After exflagellation was observed, the mice were treated intravenously (i.v.) with drug vehicle alone (10% N-methylpyrrolidnone, 40% PEG 400 in water) or 2–4 mg/kg Torin 2. Mice either received two doses 3 hrs apart or a single dose. The two dose treatment was done in triplicate, and the one dose treatment was done once. 1.5 hours after the final treatment, mice were anesthetized and 20–30 female *Anopheles stephensi* mosquitoes (6–9 days old) were allowed to feed on an infected mouse for 15 mins. Parasitemia, gametocytemia, and presence of exflagellation were examined as described previously^57^. Mosquitoes were maintained on 5% (w/v) glucose at 19°C and 80% relative humidity. At day 10 post feeding, mosquito midguts were dissected and parasite infection was measured by staining mosquito midguts with 0.2% mercurochrome and counting the numbers of oocysts per midgut. Statistical significance was determined using Student's T-test when comparing two treatment groups and One-Way ANOVA when comparing three groups. All animal experiments were done at Loyola University Chicago, in compliance with the guidelines of their Institutional Animal Care and Use Committee.

### Data analysis

The primary screen data was analyzed using customized software developed internally^58^. IC_50_ values were calculated using the Prism software (Graphpad Software, Inc. San Diego, CA). Data were presented as means ± SEM with n = 3 independent experiments.

## Author Contributions

K.C.W., W.Z., T.Q.T. and W.S. designed the study. W.S., T.Q.T., C.T.M., W.W.H., R.H. and D.J.S. performed the experiments, and collected and analyzed the data. W.S., T.Q.T., R.H., K.C.W. and W.Z. wrote the manuscript. N.S., R.H. and C.T.M. performed statistical analysis. J.C.M. aided study design. All authors discussed the results and commented on the manuscript.

## Supplementary Material

Supplementary Information SI_Gametocyte (Zheng)

## Figures and Tables

**Table 1:** Compounds with potent activity against *P. falciparum* 3D7 gametocytes

Compound Name	Gametocyte IC_50_ (μM)	Function class	Primary activity
NSC174938	0.003	Tyrosyl-DNA phosphodiesterase	Anticancer
Torin 2*	0.008	mTORC1 Inhibitor	Anticancer
Carfilzomib*	0.012	Proteasome inhibitor	Anticancer
Dactinomycin*	0.015	Transcription inhibitor	Anticancer, antibacterial
NVP-AUY922	0.047	Heat Shock Protein 90 (HSP90) Inhibitor	Anticancer
Maduramicin	0.047	Ionophore	Antiprotozoal
Narasin*	0.050	Ionophore	Antiprotozoal, antibacterial
Artesunate*	0.059	Alkylation of heme	Amebicides, Antimalarials
Artemether*	0.073	Alkylation of heme	Antimalarial
Alvespimycin	0.074	Heat Shock Protein 90 (hsp90) Inhibitor	Anticancer
Artenimol (DHA) *	0.077	Alkylation of heme	Antimalarials
Omacetaxine	0.083	Protein translation inhibitor	Anticancer
Thiram*	0.083	Metabolic poisons	Antifungal
Zinc pyrithione*	0.093	Copper import and iron–sulphur proteins	Antifungal
Phanquinone*	0.109	S-adenosylhomocysteine hydrolase	Antibacterial, antimalarial
Bortezomib*	0.118	Proteasome Inhibitor	Anticancer
Artemisinin*	0.148	Alkylation of heme	Antimalarials
Salinomycin sodium*	0.194	Ionophore	Antibacterial, antiprotozoal
Monensin sodium*	0.254	Ionophore	Antimalarial, antiprotozoal
Dipyrithione	0.263	Membrane transport inhibitor	Antibacterial, antifungal
Dicyclopentamethylenethiuram disulfide*	0.274	Monoglyceride lipase (MGL) inhibitor	Other
Methylene blue*	0.307	Monoamine oxidase inhibitor	Antimalaria, Anticancer
Quinine hemisulfate*	0.345	Hemozoin biocrystallization inhibitor	Antimalarial, analgesic, antiinflammatory
YM155	0.372	Survivin inhibitor	Anticancer
Withaferin A	0.372	NF-kappaB Activation Inhibitor	Anticancer
Adriamycin*	0.526	DNA synthesis inhibitor	Anticancer
Romidepsin	0.637	Histone deacetylase (HDAC) inhibitor	Anticancer
AZD-1152-HQPA	0.743	Aurora kinase inhibitor	Anticancer
CAY10581	0.743	Indoleamine 2,3-dioxygenase inhibitor	Anticancer
Mefloquine*	0.833	Heme polymerase inhibitor	Antimalarial, antiinflammatory
Plicamycin*	0.833	RNA synthesis inhibitor	Antibiotics, anticancer
CUDC-101	0.833	Multi target Inhibitor of HDA),EGFR/ErbB1, and HER2/neu or ErbB2	Anticancer
Auranofin*	0.935	Mitochondrial thioredoxin reductase (TrxR) inhibitor	Antirheumatic, antiinflammatory
Trametinib	0.935	Mitogen-activated protein kinase kinase (MEK MAPK/ERK kinase) inhibitor	Anticancer
GSK-458	0.935	PI3K inhibitor	Anticancer
Afatinib	0.935	Dual receptor tyrosine kinase (RTK) inhibitor	Anticancer
Panobinostat	0.935	Selective histone deacetylase inhibitor (HDAC)	Anticancer
Puromycin*	1.049	Transcription inhibitor	Antibiotic, antibacterial
Primaquine*	1.262	Not clear	Antimalarial

Note: mean IC_50_, mean half-maximum inhibitory concentrations determined from at least 3 independent experiments against *P. falciparum* 3D7 gametocyte; * indicates compounds with previously reported activities against asexual parasites. means compounds with previously reported activities against gametocytes (references are in supplementary information).

**Table 2:** Three potential protein targets in gametocytes for Torin 2

			Pull-down		DARTS		
Name of protein	Accession Number	Molecular Weight	Positive	Negative	Positive	Negative	Function
Phosphoribosyl pyrophosphate synthetase (Ribose-phosphate diphosphokinase)	PF3D7_1325100	49 kDa	4	0	6	0	Biosynthesis of purine, pyrimidine and pyridine nucleotides
Aspartate carbamoyltransferase (ATCase)	PF3D7_1344800	43 kDa	2	0	4	0	Biosynthesis of pyrimidine
Transporter, putative	PF3D7_0914700	58 kDa	3	0	1	0	Transporter

Note: Protein bands in both positive (Torin 2 affinity precipitation) and negative (Torin 1 affinity precipitation) samples were destained, reduced, and digested for mass spectrum analysis. The mass spectrum data were analyzed by SEQUEST using plasmoDB genomic database. In DARTS assays, upon pronase treatment, protein bands in positive (Torin 2 protected) and negative (Torin 1 protected) samples were processed similar as in above affinity precipitation methods. Phosphoribosyl pyrophosphate synthetase, aspartate carbamoyltransferase, and transporter are the three proteins identified by both pull-down and DARTS.
