# Supplementary material for: Chemical signatures and new drug targets for gametocytocidal drug development
Source: Sci Rep. 2014 Jan 17;4:3743. doi: 10.1038/srep03743 (PMC3894558; doi:10.1038/srep03743)
Supplement: Supplementary Information — SI_Gametocyte (Zheng) [file srep03743-s1.pdf]

Supplementary Information For:

Chemical signatures and new drug targets for gametocytocidal drug development

Wei Sun[1*], Takeshi Tanaka[2*], Crystal T. Magle[3], Wenwei Huang[1], Noel Southall[1], Ruili Huang[1], Seameen J. Dehdashti[1], John C. McKew[1], Kim C. Williamson[2,3†] and Wei Zheng[1†]

[1]National Center for Advancing Translational Sciences, National Institutes of Health, Bethesda, MD 20892, United States

[2]Laboratory of Malaria and Vector Research, National Institute of Allergy and Infectious Diseases, National Institutes of Health, Bethesda, MD 20892, United States

[3]Department of Biology, Loyola University Chicago, Chicago, IL 60660, United States

[*]These authors contributed equally to this work.

[†]To whom correspondence should be addressed:

Kim C. Williamson, PhD, Loyola University Chicago, Department of Biology 1032 W. Sheridan Rd. Chicago, IL 60660 United States Tel.: 773-508-3631; fax: 773-508-3646, Email: kwilli4@luc.edu

Wei Zheng, PhD. National Center for Advancing Translational Sciences, National Institutes of Health, 9800 Medical Center Dr. MSC 3370, Bethesda, MD 20892. Tel. 301-217-5251; Email: wzheng@mail.nih.gov

Running title: Repurposing screen of *P. falciparum* gametocytes

Keywords: malaria, gametocytes, asexual stages, drug repurposing screen, chemoinformatics, gametocytocidal agents

Supplementary Methods

Mass spectrum analysis. The excised gel bands were destained in a 1:1 mixture of $K_3Fe(CN)_6$ (30 mM) and $Na_2S_2O_3$ (100 mM), dehydrated in acetonitrile, reduced with DTT (50 mM), alkylated by iodoacetamide (120 mM), and digested with sequencing grade trypsin overnight at 37 ºC [1]. The digested peptides were extracted with 1% formic acid and subjected to liquid chromatography-tandem mass spectrometry (LC-MS/MS) analysis by Agilent 1100 nanoflow LC system coupled on-line with hybrid linear ion trap-FT-ICR instrument (LTQ-FT, Thermo Electron, San Jose, CA)[2]. Tandem MS data were used to search plasmoDB genomic database (http://www.plasmodb.org) using SEQUEST. Positive hits were re-evaluated by Scaffold (Proteome Softwares, Inc., Portland, OR) and hits that showed 95% probability in Scaffold were considered as significant hits.

SI Table 1, Cytotoxicity of gametocytocidal compounds in mammalian HepG2 cell line.

Compound	IC_{50} (μM) in HepG2 cells	% Max response
NSC174938	Inactive	13
Torin 2	9.350	-46
Carfilzomib	1.177	-79
Dactinomycin	Inactive	1
NVP-AUY922	0.148	-88
Maduramicin	37.221	-42
Narasin	27.041	-98
Artesunate	Inactive	18
Artemether	Inactive	7
Alvespimycin	0.118	-94
Artenimol (DHA)	Inactive	20
Omacetaxine	Curve not complete	-27
Thiram	Inactive	23
Zinc pyrithione	2.148	-99
Phanquinone	Inactive	23
Bortezomib	0.148	-82

Artemisinin	Inactive	18
Salinomycin sodium	29.566	-69
Monensin sodium	Inactive	40
Dipyrithione	10.765	-93
Dicyclopentamethylenethiuram disulfide	Inactive	0
Methylene blue	Inactive	-20
Quinine hemisulfate	Inactive	2
YM155	4.686	-92
Withaferin A	9.350	-80
Adriamycin	Inactive	-35
Romidepsin	0.074	-98
AZD-1152-HQPA	Inactive	11
CAY10581	17.062	-86
Mefloquine	29.566	-97
Plicamycin	Inactive	3
CUDC-101	6.793	-76
Auranofin	9.350	-98
Trametinib	Inactive	14
GSK-458	Inactive	33
Afatinib	18.655	-100
Panobinostat	0.372	-74
Puromycin	11.770	-71
Primaquine	17.831	-65

Note: "Inactive": no significant activity at the highest tested compound concentration (46 µM). Each compound was examined in 11 concentrations at a 1:3 dilution against HepG2 cells.

Table 2: Gametocytocidal activity of the malaria box

Supplier ID	Curve Class	IC50(uM)	Max Resp	Sample ID
MMV020788	-1.2	0.105	-43	NCGC00273738-01
MMV665785	-1.2	0.148	-50	NCGC00273790-01
MMV665782	-1.2	0.209	-31	NCGC00273788-01
MMV020500	-1.2	0.235	-63	NCGC00273729-01
MMV000442	-1.2	0.332	-49	NCGC00273570-01
MMV006087	-1.2	0.372	-66	NCGC00054324-02
MMV665875	-1.2	0.372	-41	NCGC00273820-01
**MMV019406**	**-1.1**	**0.418**	**-82**	**NCGC00273709-01**
MMV000570	-1.2	0.469	-29	NCGC00273577-01
MMV007181	-1.2	0.590	-30	NCGC00273642-01
MMV666116	-1.2	0.662	-46	NCGC00273897-01
MMV665841	-1.2	0.662	-28	NCGC00273814-01
MMV006455	-1.2	0.743	-40	NCGC00273620-01
MMV000972	-1.4	0.743	-32	NCGC00273598-01
**MMV666125**	**-1.1**	**0.833**	**-116**	**NCGC00273900-01**
MMV019127	-1.2	0.935	-56	NCGC00273702-01
MMV006558	-1.2	0.935	-44	NCGC00273624-01
MMV085583	-1.1	1.049	-82	NCGC00273749-01
MMV666060	-1.2	1.049	-45	NCGC00273875-01
MMV665789	-1.2	1.321	-58	NCGC00273792-01
MMV000443	-1.2	1.321	-54	NCGC00273571-01
MMV084940	-1.1	1.321	-133	NCGC00273746-01
MMV006172	-1.1	1.482	-98	NCGC00167823-04
MMV000248	-1.1	1.482	-99	NCGC00273565-01
MMV666061	-1.2	1.663	-57	NCGC00273876-01
MMV665830	-1.1	1.663	-88	NCGC00273811-01
MMV019918	-1.1	1.866	-106	NCGC00273721-01
MMV000448	-1.1	1.866	-105	NCGC00245884-02
MMV666597	-1.4	2.093	-27	NCGC00273902-01
MMV000848	-1.1	2.093	-93	NCGC00273594-01
MMV020654	-1.2	2.093	-37	NCGC00273734-01
MMV001049	-1.2	2.349	-76	NCGC00273602-01
MMV667492	-1.2	2.349	-59	NCGC00273958-01
MMV009063	-1.1	2.349	-99	NCGC00273683-01
MMV073843	-1.1	2.349	-98	NCGC00273743-01
MMV667488	-1.2	2.635	-32	NCGC00273916-01
MMV007591	-1.1	2.635	-109	NCGC00273657-01
MMV006303	-1.1	2.635	-102	NCGC00273614-01

MMV000356	-1.2	2.635	-72	NCGC00273569-01
MMV665878	-1.1	2.635	-82	NCGC00273822-01
MMV667491	-1.1	2.635	-95	NCGC00273957-01
MMV006787	-1.1	2.635	-94	NCGC00273629-01
MMV019017	-1.1	2.635	-94	NCGC00273698-01
MMV019881	-1.1	2.957	-90	NCGC00273720-01
MMV665882	-2.1	2.957	-97	NCGC00273825-01
MMV665971	-1.2	2.957	-40	NCGC00273862-01
MMV396693	-1.1	2.957	-85	NCGC00273939-01
MMV665929	-1.2	2.957	-73	NCGC00273847-01
MMV396794	-1.1	2.957	-106	NCGC00273950-01
MMV020505	-1.1	3.317	-98	NCGC00273730-01
MMV396744	-1.2	3.317	-70	NCGC00273948-01
MMV006429	-1.1	3.317	-82	NCGC00273619-01
MMV665806	-1.1	3.317	-89	NCGC00273801-01
MMV001038	-1.2	3.317	-71	NCGC00273600-01
MMV008956	-1.2	3.317	-73	NCGC00273680-01
MMV396703	-1.1	3.722	-81	NCGC00273940-01
MMV665820	-1.1	3.722	-101	NCGC00273808-01
MMV665902	-1.1	3.722	-99	NCGC00073789-02
MMV665809	-1.2	3.722	-58	NCGC00273803-01
MMV011576	-1.1	3.722	-98	NCGC00273692-01
MMV000760	-1.1	3.722	-91	NCGC00273591-01
MMV667490	-1.1	4.176	-79	NCGC00273956-01
MMV019780	-1.1	4.176	-106	NCGC00273718-01
MMV665805	-1.2	4.176	-69	NCGC00273800-01
MMV000662	-1.1	4.176	-81	NCGC00273586-01
MMV000478	-1.2	4.176	-76	NCGC00273573-01
MMV019555	-1.1	4.686	-104	NCGC00273710-01
MMV306025	-1.1	4.686	-98	NCGC00273923-01
MMV011438	-1.1	5.258	-89	NCGC00273690-01
MMV666109	-1.2	5.258	-58	NCGC00273895-01
MMV007285	-1.2	5.258	-38	NCGC00273649-01
MMV000483	-1.1	5.258	-82	NCGC00273575-01
MMV019995	-1.1	5.258	-99	NCGC00273722-01
MMV000481	-1.1	5.258	-81	NCGC00273574-01
MMV665906	-1.2	5.258	-61	NCGC00273835-01
MMV305841	-1.1	5.258	-95	NCGC00273754-01
MMV665812	-1.2	5.258	-74	NCGC00273804-01
MMV665948	-1.4	5.899	-52	NCGC00273857-01
MMV011795	-2.2	5.899	-53	NCGC00273693-01

MMV665941	-1.1	5.899	-118	NCGC00273853-01
MMV665928	-2.2	5.899	-78	NCGC00273846-01
MMV007474	-1.1	5.899	-93	NCGC00273652-01
MMV019690	-1.1	6.619	-89	NCGC00273713-01
MMV665944	-2.1	6.619	-96	NCGC00273855-01
MMV000986	-1.1	6.619	-93	NCGC00273599-01
MMV396797	-1.1	6.619	-90	NCGC00273951-01
MMV666689	-1.2	6.619	-64	NCGC00273911-01
MMV665831	-2.2	7.427	-58	NCGC00273812-01
MMV007092	-1.1	7.427	-94	NCGC00273637-01
MMV007907	-2.2	7.427	-73	NCGC00273669-01
MMV000445	-1.1	7.427	-110	NCGC00273572-01
MMV666604	-1.2	7.427	-68	NCGC00273906-01
MMV000604	-2.2	7.427	-55	NCGC00273578-01
MMV085203	-2.1	7.427	-97	NCGC00273747-01
MMV009015	-2.2	7.427	-74	NCGC00273681-01
MMV006203	-1.1	8.333	-94	NCGC00273611-01
MMV000788	-1.1	8.333	-97	NCGC00273592-01
MMV000787	-1.1	8.333	-98	NCGC00114964-02
MMV000444	-1.1	8.333	-107	NCGC00101833-02
MMV000839	-1.1	8.333	-97	NCGC00273593-01
MMV665949	-1.2	9.350	-40	NCGC00246301-03
MMV396632	-2.2	9.350	-76	NCGC00273926-01
MMV665817	-2.1	9.350	-107	NCGC00273807-01
MMV665814	-2.1	9.350	-107	NCGC00273806-01
MMV666080	-2.2	9.350	-75	NCGC00273885-01
MMV007557	-2.2	9.350	-72	NCGC00273653-01
MMV000620	-2.2	9.350	-78	NCGC00273581-01
MMV000642	-2.2	9.350	-75	NCGC00273583-01
MMV000963	-2.1	9.350	-86	NCGC00273597-01
MMV007020	-2.1	10.490	-102	NCGC00273635-01
MMV666069	-2.1	10.490	-86	NCGC00273879-01
MMV000634	-2.1	10.490	-84	NCGC00121681-02
MMV666023	-2.1	10.490	-89	NCGC00273870-01
MMV396770	-2.1	10.490	-93	NCGC00273949-01
MMV006169	-2.2	11.770	-80	NCGC00273609-01
MMV006962	-2.1	11.770	-94	NCGC00273634-01
MMV665969	-2.2	11.770	-76	NCGC00273861-01
MMV665980	-2.1	11.770	-81	NCGC00273866-01
MMV396669	-2.2	11.770	-75	NCGC00273933-01
MMV006427	-2.2	11.770	-74	NCGC00273618-01

MMV128432	-2.1	11.770	-81	NCGC00273751-01
MMV001246	-2.2	11.770	-72	NCGC00273605-01
MMV020243	-2.2	11.770	-73	NCGC00273723-01
MMV019266	-2.2	11.770	-72	NCGC00273707-01
MMV396633	-2.2	11.770	-72	NCGC00273927-01
MMV665864	-2.1	13.207	-93	NCGC00273818-01
MMV000720	-2.1	13.207	-112	NCGC00273589-01
MMV665857	-2.2	14.818	-77	NCGC00273817-01
MMV011522	-2.2	14.818	-73	NCGC00273691-01
MMV665946	-2.2	14.818	-77	NCGC00273856-01
MMV665979	-2.1	14.818	-89	NCGC00273865-01
MMV011944	-2.2	14.818	-68	NCGC00273696-01
MMV666067	-2.1	14.818	-95	NCGC00273878-01
MMV006764	-2.2	14.818	-76	NCGC00273628-01
MMV665888	-2.2	14.818	-76	NCGC00273827-01
MMV020549	-2.2	14.818	-75	NCGC00273732-01
MMV006656	-2.2	14.818	-72	NCGC00273625-01
MMV666687	-2.1	14.818	-100	NCGC00273909-01
MMV009085	-2.2	14.818	-71	NCGC00273684-01
MMV665786	-2.1	14.818	-89	NCGC00273791-01
MMV008160	-2.1	14.818	-87	NCGC00273673-01
MMV396723	-2.1	14.818	-92	NCGC00273945-01
MMV001041	-2.2	14.818	-63	NCGC00273601-01
MMV666022	-2.1	14.818	-83	NCGC00140877-02
MMV665961	-2.1	16.626	-95	NCGC00273860-01
MMV665927	-2.1	16.626	-88	NCGC00273845-01
MMV396715	-2.1	16.626	-84	NCGC00273942-01
MMV006513	-2.1	16.626	-85	NCGC00273622-01
MMV008455	-2.1	16.626	-89	NCGC00273677-01
MMV000304	-2.1	16.626	-95	NCGC00273566-01
MMV666054	-2.1	16.626	-125	NCGC00273873-01
MMV396663	-2.1	16.626	-85	NCGC00273930-01
MMV666079	-2.1	16.626	-81	NCGC00273884-01
MMV665953	-2.1	18.655	-94	NCGC00273858-01
MMV665794	-2.1	18.655	-98	NCGC00273793-01
MMV396749	-2.2	18.655	-61	NCGC00071499-03
MMV006861	-2.2	18.655	-63	NCGC00176882-02
MMV665940	2.2	18.655	46	NCGC00273852-01
MMV403679	-2.2	18.655	-64	NCGC00273952-01
MMV665810	-2.2	18.655	-78	NCGC00174046-03
MMV665886	2.2	18.655	44	NCGC00273826-01

MMV011895	-2.2	18.655	-78	NCGC00273695-01
MMV019110	-2.2	18.655	-65	NCGC00273700-01
MMV000753	-2.2	18.655	-68	NCGC00273590-01
MMV665909	-2.2	18.655	-65	NCGC00249404-02
MMV007224	-2.1	20.931	-96	NCGC00273645-01
MMV665807	-2.1	20.931	-84	NCGC00273802-01
MMV007363	-2.2	23.485	-75	NCGC00062758-02
MMV020403	-2.2	23.485	-73	NCGC00273725-01
MMV006825	-2.2	23.485	-74	NCGC00273631-01
MMV396736	-2.2	23.485	-70	NCGC00273947-01
MMV396717	-2.2	23.485	-70	NCGC00273943-01
MMV000704	-2.2	23.485	-69	NCGC00273588-01
MMV274073	-2.2	23.485	-66	NCGC00273922-01
MMV007875	-2.2	23.485	-72	NCGC00273666-01
MMV007617	-2.2	23.485	-70	NCGC00273658-01
MMV020660	-2.2	23.485	-67	NCGC00273735-01
MMV665852	-2.1	23.485	-88	NCGC00273816-01
MMV020912	-2.2	23.485	-63	NCGC00273740-01
MMV396719	-2.2	23.485	-71	NCGC00273944-01
MMV019199	-2.2	23.485	-66	NCGC00273703-01
MMV000917	-2.2	29.566	-63	NCGC00273596-01
MMV080034	-2.2	29.566	-61	NCGC00073206-02
MMV009127	-2.2	29.566	-72	NCGC00273686-01
MMV007273	-2.2	29.566	-62	NCGC00273647-01
MMV008212	-2.2	29.566	-64	NCGC00273674-01
MMV019738	-2.2	29.566	-61	NCGC00273715-01
MMV666110	-2.2	29.566	-73	NCGC00273896-01
MMV011832	-2.2	29.566	-67	NCGC00273694-01
MMV665972	-2.2	29.566	-62	NCGC00273863-01
MMV665943	-2.2	29.566	-69	NCGC00273854-01
MMV006522	-2.2	29.566	-59	NCGC00273623-01
MMV006704	-2.2	29.566	-79	NCGC00273626-01
MMV020490	-2.2	29.566	-61	NCGC00273727-01
MMV006319	-2.2	29.566	-60	NCGC00273616-01
MMV666071	-2.2	29.566	-71	NCGC00273881-01
MMV666102	-2.2	29.566	-75	NCGC00273890-01
MMV666692	-2.2	29.566	-54	NCGC00273913-01
MMV000699	-2.2	29.566	-70	NCGC00273587-01
MMV665897	-2.2	29.566	-66	NCGC00273831-01
MMV019746	-2.2	29.566	-74	NCGC00273717-01
MMV666057	-2.2	29.566	-57	NCGC00273874-01

MMV666607	-2.2	29.566	-57	NCGC00273907-01
MMV665803	-2.2	29.566	-58	NCGC00273799-01
MMV665914	-2.2	29.566	-63	NCGC00273838-01
MMV666020	-2.2	37.221	-64	NCGC00273869-01
MMV396704	-2.2	37.221	-57	NCGC00273941-01
MMV007384	-2.2	37.221	-59	NCGC00273650-01
MMV020750	-2.2	37.221	-55	NCGC00273737-01
MMV006767	-2.2	37.221	-53	NCGC00031873-02
MMV665796	-2.2	37.221	-58	NCGC00273794-01
MMV008416	-2.2	37.221	-53	NCGC00073772-02
MMV000621	-2.2	37.221	-59	NCGC00273582-01
MMV019741	-2.2	37.221	-65	NCGC00273716-01
MMV666025	-2.2	37.221	-60	NCGC00273871-01
MMV000561	-2.2	Inactive	-52	NCGC00108865-02
MMV665826	-2.2	Inactive	-52	NCGC00273810-01
MMV666686	-2.2	Inactive	-50	NCGC00273908-01
MMV006587	-2.2	Inactive	-49	NCGC00019462-03
MMV020548	-3	Inactive	-56	NCGC00273731-01
MMV665915	-2.2	Inactive	-44	NCGC00273839-01
MMV006278	-2.2	Inactive	-52	NCGC00273613-01
MMV665876	-2.2	Inactive	-47	NCGC00273821-01
MMV007041	-2.2	Inactive	-50	NCGC00273636-01
MMV665850	-2.2	Inactive	-50	NCGC00273815-01
MMV000653	-2.2	Inactive	-49	NCGC00273585-01
MMV000340	-2.2	Inactive	-49	NCGC00273568-01
MMV666108	-2.2	Inactive	-51	NCGC00273894-01
MMV666070	-2.2	Inactive	-47	NCGC00273880-01
MMV007564	-2.2	Inactive	-54	NCGC00273654-01
MMV000326	-2.2	Inactive	-52	NCGC00273567-01
MMV665797	-2.2	Inactive	-47	NCGC00273795-01
MMV665824	-2.4	Inactive	-53	NCGC00273809-01
MMV665783	-2.2	Inactive	-50	NCGC00273789-01
MMV000619	-2.2	Inactive	-51	NCGC00273580-01
MMV007275	-2.2	Inactive	-41	NCGC00273648-01
MMV020275	-2.2	Inactive	-48	NCGC00273724-01
MMV019064	-2.2	Inactive	-42	NCGC00273699-01
MMV001239	-2.2	Inactive	-40	NCGC00273603-01
MMV006882	-2.2	Inactive	-43	NCGC00273632-01
MMV000648	-2.2	Inactive	-46	NCGC00273584-01
MMV019313	-2.2	Inactive	-46	NCGC00273708-01
MMV011436	-2.2	Inactive	-49	NCGC00273689-01

MMV001241	-2.2	Inactive	-48	NCGC00273604-01
MMV007764	-2.2	Inactive	-41	NCGC00273662-01
MMV665800	-2.2	Inactive	-48	NCGC00273798-01
MMV666075	-2.4	Inactive	-34	NCGC00273883-01
MMV665890	-3	Inactive	-38	NCGC00273828-01
MMV666105	-2.2	Inactive	-42	NCGC00273892-01
MMV019871	-2.2	Inactive	-42	NCGC00273719-01
MMV019758	-3	Inactive	-32	NCGC00165085-02
MMV396672	-2.2	Inactive	-37	NCGC00273934-01
MMV665901	-2.4	Inactive	-41	NCGC00056290-02
MMV665799	4	Inactive	-28	NCGC00273797-01
MMV665881	4	Inactive	-29	NCGC00273824-01
MMV019124	-2.4	Inactive	-34	NCGC00273701-01
MMV019241	-2.4	Inactive	-32	NCGC00273705-01
MMV000911	-2.2	Inactive	-36	NCGC00273595-01
MMV019074	-2.2	Inactive	-32	NCGC00103844-02
MMV008474	-2.4	Inactive	-32	NCGC00273678-01
MMV666124	-3	Inactive	-33	NCGC00273899-01
MMV665898	4	Inactive	-26	NCGC00273832-01
MMV020492	-2.2	Inactive	-36	NCGC00273728-01
MMV666009	-2.4	Inactive	-36	NCGC00061228-02
MMV084434	4	Inactive	-24	NCGC00273745-01
MMV665913	4	Inactive	-15	NCGC00273837-01
MMV008829	4	Inactive	-26	NCGC00273679-01
MMV000617	-2.4	Inactive	-31	NCGC00273579-01
MMV075490	-2.2	Inactive	-38	NCGC00273744-01
MMV001230	4	Inactive	-26	NCGC00136467-02
MMV666106	4	Inactive	-29	NCGC00273893-01
MMV665883	4	Inactive	-25	NCGC00056971-02
MMV666601	4	Inactive	-26	NCGC00273905-01
MMV020651	4	Inactive	-23	NCGC00273733-01
MMV665924	4	Inactive	-23	NCGC00273844-01
MMV019662	4	Inactive	-21	NCGC00273711-01
MMV007839	4	Inactive	-23	NCGC00273665-01
MMV396665	4	Inactive	-24	NCGC00273932-01
MMV665899	4	Inactive	-26	NCGC00273833-01
MMV007208	4	Inactive	-28	NCGC00273644-01
MMV665894	4	Inactive	-23	NCGC00273830-01
MMV020885	4	Inactive	-29	NCGC00273739-01
MMV396635	4	Inactive	-24	NCGC00273928-01
MMV665840	4	Inactive	-16	NCGC00188060-02

MMV000498	4	Inactive	-20	NCGC00104250-02
MMV001318	4	Inactive	-20	NCGC00273607-01
MMV665879	4	Inactive	-23	NCGC00273823-01
MMV665827	4	Inactive	7	NCGC00065006-02
MMV666062	4	Inactive	12	NCGC00273877-01
MMV665874	4	Inactive	-2	NCGC00273819-01
MMV007686	4	Inactive	-2	NCGC00273660-01
MMV001255	4	Inactive	2	NCGC00273606-01
MMV396680	4	Inactive	-21	NCGC00273937-01
MMV667486	4	Inactive	-4	NCGC00073726-03
MMV498479	4	Inactive	28	NCGC00273785-01
MMV006753	4	Inactive	3	NCGC00273627-01
MMV396681	4	Inactive	-3	NCGC00273938-01
MMV006706	4	Inactive	-18	NCGC00021112-04
MMV665918	4	Inactive	-14	NCGC00273842-01
MMV009060	4	Inactive	20	NCGC00273682-01
MMV019066	4	Inactive	-11	NCGC00045882-03
MMV008138	4	Inactive	-1	NCGC00273672-01
MMV396664	4	Inactive	-4	NCGC00273931-01
MMV007113	4	Inactive	-5	NCGC00273638-01
MMV396595	4	Inactive	7	NCGC00273925-01
MMV007374	4	Inactive	-9	NCGC00083570-02
MMV006913	4	Inactive	0	NCGC00273633-01
MMV009108	4	Inactive	-14	NCGC00273685-01
MMV666095	4	Inactive	28	NCGC00273888-01
MMV666072	4	Inactive	12	NCGC00273882-01
MMV011099	4	Inactive	-5	NCGC00020041-03
MMV666691	4	Inactive	-7	NCGC00273912-01
MMV007577	4	Inactive	18	NCGC00118500-02
MMV666123	4	Inactive	-6	NCGC00273898-01
MMV667489	4	Inactive	1	NCGC00273955-01
MMV665836	4	Inactive	13	NCGC00273813-01
MMV665813	4	Inactive	-14	NCGC00273805-01
MMV667487	4	Inactive	-4	NCGC00273915-01
MMV665917	4	Inactive	-1	NCGC00273841-01
MMV007977	4	Inactive	25	NCGC00273670-01
MMV006250	4	Inactive	3	NCGC00273612-01
MMV665939	4	Inactive	-3	NCGC00273851-01
MMV666081	4	Inactive	23	NCGC00273886-01
MMV665977	4	Inactive	2	NCGC00273864-01
MMV006457	4	Inactive	14	NCGC00273621-01

MMV020439	4	Inactive	-25	NCGC00273726-01
MMV666021	4	Inactive	-4	NCGC00075146-02
MMV085471	4	Inactive	1	NCGC00273748-01
MMV007228	4	Inactive	-1	NCGC00273646-01
MMV086103	4	Inactive	0	NCGC00273750-01
MMV665987	4	Inactive	0	NCGC00273867-01
MMV007127	4	Inactive	7	NCGC00273640-01
MMV396594	4	Inactive	-12	NCGC00273924-01
MMV007199	4	Inactive	-2	NCGC00273643-01
MMV056726	4	Inactive	-5	NCGC00273742-01
MMV007430	4	Inactive	29	NCGC00273651-01
MMV008127	4	Inactive	-5	NCGC00273671-01
MMV665843	4	Inactive	-7	NCGC00267443-02
MMV007791	4	Inactive	1	NCGC00273663-01
MMV011259	4	Inactive	4	NCGC00273921-01
MMV666596	4	Inactive	14	NCGC00273901-01
MMV006937	4	Inactive	22	NCGC00079563-03
MMV006188	4	Inactive	19	NCGC00273610-01
MMV666688	4	Inactive	-20	NCGC00273910-01
MMV007571	4	Inactive	6	NCGC00273655-01
MMV019258	4	Inactive	-8	NCGC00273706-01
MMV007116	4	Inactive	32	NCGC00273639-01
MMV007396	4	Inactive	23	NCGC00118502-02
MMV020942	4	Inactive	-19	NCGC00273741-01
MMV007808	4	Inactive	-9	NCGC00273664-01
MMV007978	4	Inactive	-13	NCGC00241415-02
MMV007881	4	Inactive	15	NCGC00273667-01
MMV666599	4	Inactive	4	NCGC00273903-01
MMV638723	4	Inactive	-5	NCGC00273953-01
MMV008294	4	Inactive	-8	NCGC00273676-01
MMV665935	4	Inactive	25	NCGC00273849-01
MMV008173	4	Inactive	0	NCGC00138289-02
MMV006389	4	Inactive	8	NCGC00273617-01
MMV007654	4	Inactive	-3	NCGC00273659-01
MMV665923	4	Inactive	14	NCGC00273843-01
MMV665936	4	Inactive	5	NCGC00273850-01
MMV007906	4	Inactive	12	NCGC00273668-01
MMV019670	4	Inactive	13	NCGC00273712-01
MMV665891	4	Inactive	18	NCGC00273829-01
MMV000563	4	Inactive	19	NCGC00273576-01
MMV019762	4	Inactive	-1	NCGC00188067-02

MMV019202	4	Inactive	-23	NCGC00273704-01
MMV007160	4	Inactive	-3	NCGC00273641-01
MMV666600	4	Inactive	2	NCGC00273904-01
MMV142383	4	Inactive	-10	NCGC00273752-01
MMV007695	4	Inactive	17	NCGC00273661-01
MMV666093	4	Inactive	-10	NCGC00273887-01
MMV645672	4	Inactive	3	NCGC00273954-01
MMV665994	4	Inactive	5	NCGC00273868-01
MMV007574	4	Inactive	12	NCGC00273656-01
MMV001344	4	Inactive	-13	NCGC00273608-01
MMV665908	4	Inactive	-26	NCGC00273836-01
MMV396705	4	Inactive	-1	NCGC00020852-04
MMV008270	4	Inactive	26	NCGC00273675-01
MMV665954	4	Inactive	5	NCGC00273859-01
MMV019700	4	Inactive	17	NCGC00273714-01
MMV020700	4	Inactive	-2	NCGC00273736-01
MMV011567	4	Inactive	1	NCGC00117107-02
MMV665916	4	Inactive	-1	NCGC00273840-01
MMV396679	4	Inactive	0	NCGC00273936-01
MMV011256	4	Inactive	-9	NCGC00273687-01
MMV008149	4	Inactive	17	NCGC00128966-02
MMV666693	4	Inactive	19	NCGC00273914-01
MMV666101	4	Inactive	1	NCGC00273889-01
MMV396678	4	Inactive	4	NCGC00273935-01
MMV006309	4	Inactive	2	NCGC00273615-01
MMV666103	4	Inactive	-11	NCGC00273891-01
MMV396652	4	Inactive	-1	NCGC00273929-01
MMV666026	4	Inactive	0	NCGC00273872-01
MMV396726	4	Inactive	-1	NCGC00273946-01
MMV665934	4	Inactive	17	NCGC00273848-01
MMV665798	4	Inactive	-7	NCGC00273796-01
MMV018984	4	Inactive	14	NCGC00273697-01
MMV006820	4	Inactive	24	NCGC00273630-01
MMV665904	4	Inactive	-7	NCGC00273834-01

Note: Each compound was examined in 11 concentrations at a 1:3 dilution against stages III-V 3D7 gametocytes. Compounds showing IC_{50} less than 1 μM and activity <-75% were highlighted. DMSO activity was set at 0%. 100 nM epoxomicin activity was set at -100%. Curve class is defined as the following: data with the highest confidence are those in curve class 1.1. Active compounds are those in curve class 1.1, 1.2, 2.1 and possibly 2.2. Curve class 4 compounds are considered inactive, with IC_{50} estimated to be greater than twice the highest concentration tested. The remaining curve classes are less confident or inconclusive.

SI Table 3. Structure clusters enriched with gametocyte selective compounds.

Compound name	Cluster (row. column)	Activity against gametocytes			Activity against asexual parasites		
		Enrichment (-LogP)	IC_{50} (μM)	Efficacy (% response)	Enrichment (-LogP)	IC_{50} (μM)	Efficacy (% response)
Clomipramine hydrochloride	14. 10	5.07	3.55	-84.02	-0.32	inactive	inactive
Imipramine hydrochloride	14. 10	5.07	7.08	-91.53	-0.32	inactive	inactive
Desipramine hydrochloride	14. 10	5.07	12.59	-101.19	-0.32	inactive	inactive
Lofepramine hydrochloride	14. 10	5.07	19.95	-102.88	-0.32	10.00	-103.99
Dimetacrine	14. 10	5.07	22.39	-76.95	-0.32	inactive	inactive
Trimipramine maleate	14. 10	5.07	22.39	-46.95	-0.32	30.73	-46.68
Imipramine	14. 10	5.07	25.12	-81.59	-0.32	inactive	inactive
Clomipramine	14. 10	5.07	35.48	-117.76	-0.32	inactive	inactive
Desipramine hydrochloride	14. 10	5.07	35.48	-81.86	-0.32	inactive	inactive
Trimipramine maleate	14. 10	5.07	inactive	inactive	-0.32	inactive	inactive
Oxcarbazepine	14. 10	5.07	inactive	inactive	-0.32	inactive	inactive
Lumefantrine	16. 14	7.92	0.00	-76.51	0.00	0.02	-93.57
Cyproheptadine hydrochloride	16. 14	7.92	3.55	-80.99	0.00	inactive	inactive
Cyclobenzaprine hydrochloride	16. 14	7.92	5.62	-94.80	0.00	inactive	inactive
Amitriptyline hydrochloride	16. 14	7.92	6.31	-102.06	0.00	11.22	-82.37
Cyclobenzaprine hydrochloride	16. 14	7.92	10.00	-100.40	0.00	inactive	inactive
Protriptyline hydrochloride	16. 14	7.92	12.59	-81.72	0.00	inactive	inactive
Nortriptyline hdrochloride	16. 14	7.92	22.39	-110.69	0.00	inactive	inactive
Amitriptyline hydrochloride	16. 14	7.92	35.48	-126.71	0.00	11.22	-82.37
Cyproheptadine	16. 14	7.92	35.48	-122.81	0.00	inactive	inactive

Melitracen hydrochloride	16. 14	7.92	35.48	-103.56	0.00	inactive	inactive
Nortriptyline hydrochloride	16. 14	7.92	35.48	-102.48	0.00	inactive	inactive
Protriptyline hydrochloride	16. 14	7.92	35.48	-83.39	0.00	inactive	inactive
Metergoline	2. 14	2.09	12.59	-35.97	0.15	12.59	-71.24
Cabergoline	2. 14	2.09	17.78	-80.11	0.15	17.78	-63.65
Metergoline	2. 14	2.09	22.39	-71.10	0.15	12.59	-71.24
Pergolide methanesulfonate	2. 14	2.09	22.39	-83.45	0.15	inactive	inactive
Nicergoline	2. 14	2.09	22.39	-39.39	0.15	inactive	inactive
Terguride	2. 14	2.09	28.18	-80.68	0.15	inactive	inactive
Mesulergine	2. 14	2.09	35.48	-64.00	0.15	inactive	inactive
Pergolide methanesulfonate	2. 14	2.09	inactive	inactive	0.15	inactive	inactive

SI Table 4. Compounds selective against gametocytes over asexual parasites, against asexual parasites over gametocytes, and active against both gametocytes and asexual parasites.

Selectivity	Indications	Targets
Gametocyte	Serotonin agents<br>Anti-depression<br>Muscarinic agents<br>Adrenergic agents	Cholinergic receptor, muscarinic 2<br>5-Hydroxytryptamine (serotonin) receptor 2A<br>Cholinergic receptor, muscarinic 1<br>Solute carrier family 6 (neurotransmitter transporter, serotonin), member 4
Asexual	Dihydrofolate reductase inhibitor<br>Folic acid inhibitor	Dihydrofolate reductase
Active against both	Fungicides<br>Antiseptic<br>Topoisomerase I inhibitor	Topoisomerase (DNA) II alpha 170kDa<br>Potassium voltage-gated channel, subfamily H (eag-related), member 2

SI Table 5. Predicted target proteins based on *P. falciparum* homologs derived from the drug profiling data.

Compound Name	Function class	Pf homolog	Homolog (Blast score)	Gene ID
NVP-AUY922	Heat Shock Protein 90 (HSP90) Inhibitor	Yes	5.40E-178	708400
Alvespimycin	Heat Shock Protein 90 (HSP90) Inhibitor	Yes	"	"
Phanquinone*	S-adenosylhomocysteine hydrolase	Yes	6.60E-128	520900
Romidepsin	Histone deacetylase (HDAC) inhibitor	Yes	0	925700
Panobinostat	Selective histone deacetylase inhibitor (HDAC)	Yes	"	"
CUDC-101	Multi site Inhibitor of HDAC,EGFR/ErbB1, and HER2/neu or ErbB2	Yes	"	"
AZD-1152-HQPA	Aurora kinase inhibitor	Yes	5.00E-50	605300
Auranofin*	Mitochondrial thioredoxin reductase (TrxR) inhibitor	Yes	1.00E-48	1438900
GSK-458	PI3K inhibitor	Yes	6.00E-65	515300
Bortezomib*	Proteasome Inhibitor	Yes	4.00E-76	1011400
NSC174938	Tyrosyl-DNA phosphodiesterase	No		
Dicyclopentamethylenethiuram disulfide*	Monoglyceride lipase (MGL) inhibitor	No		
YM155	Survivin inhibitor	No		
Withaferin A	NF-kappaB Activation Inhibitor	No		
CAY10581	Indoleamine 2,3-dioxygenase inhibitor	No		
Afatinib	Dual receptor tyrosine kinase (RTK) inhibitor	No		

Note: The *P. falciparum* genomic data base, PlasmoDB (http://www.plasmodb.org) was queried for each of the predicted target proteins by annotation and using a BLAST search. The gene id for *P. falciparum* strain 3D7 is listed for genes with BLAST e-scores of <1x10^{-15}. For genes with several homologs the one with the lowest BLAST e-score is listed. The 0 means 0 probability that it is not the target in 100% confidence.

SI Table 6. Twenty gametocytocidal compounds previously reported in literatures[3]

Compound Name	Function class	Primay activity
Artesunate	Alkylation of heme	Amebicides, Antimalarials
Artenimol (DHA)	Alkylation of heme	Antimalarials
Artemether	Alkylation of heme	Antimalarial
Artemisinin	Alkylation of heme	Antimalarials
Methylene blue	Monoamine oxidase inhibitor	Antimalaria, antineoplastic
Quinine hemisulfate	Hemozoin biocrystallization inhibitor	Antimalarial, analgesic, antiinflammatory
Mefloquine	Unclear, proposed as Heme polymerase inhibitor	Antimalarial, antiinflammatory
Primaquine diphosphate	Not clear	Antimalarial
Epoxomicin	Proteasome inhibitor	Antiinflammatory
Tipranavir	Protease inhibitor	HIV infection
Thiostrepton	Protein synthesis	Antibiotic
Riboflavin	Energy metabolism	Micronutrient
Trioxaquine (DU1302)	Heme alkylation and heme stacking	Others
9-anilinoacridine	Parasite DNA topoisomerase II inhibitor	Antimalarial
Spiroindolone (NITD609)	Block protein synthesis	Antimalarial
4-aminoquinoline (TDR58846)	Hemoglobin degradation	Antimalarial
Carfilzomib	Proteasome inhibitor	Anticancer
Dactinomycin	Transcription inhibitor	Anticancer, antibacterial
Puromycin dihydrochloride	Transcription inhibitor	Antibiotic, antibacterial
Torin-2	mTORC1 Inhibitor	Anticancer

SI Table 7. Results of compound profiling against *P. falciparum* strains 3D7, HB3 and Dd2 gametocytes.

Compound name	3D7-gametocytes	HB3-gametocytes	Dd2-gametocytes
	IC_{50}(μM)	IC_{50}(μM)	IC_{50}(μM)
Panobinostat	0.935	0.148	0.118
CUDC-101	0.833	0.152	0.429
Carfilzomib	0.004	0.003	0.002
Torin-2	0.008	0.015	0.012
Dactinomycin	0.015	0.019	0.033
Maduramicin ammonium	0.024	0.012	0.037
NVP-AUY922	0.047	0.042	0.047
Narasin	0.050	0.136	0.076
Artesunate	0.059	0.047	0.030
Omacetaxine mepesuccinate	0.059	0.017	0.037
Lumefantrine	0.059	0.033	0.013
Mefloquine hydrochloride	0.074	0.059	0.053
Artemether	0.074	0.047	0.053
Alvespimycin	0.074	0.235	0.094
Artenimol	0.077	0.059	0.053
Thiram	0.083	0.148	0.148
Zinc pyrithione	0.093	0.059	0.059
Tetraethylthiuram disulfide	0.096	0.148	0.296
Disulfiram	0.096	0.108	0.096
Phanquinone	0.109	0.037	0.053
Salinomycin sodium	0.118	0.372	0.296
Bortezomib	0.118	0.074	0.094
Diphenyleneiodonium	0.132	0.296	0.209
Artemisinin	0.148	0.074	0.061
Salinomycin monosodium	0.194	0.469	0.296
Chloroquine diphosphate	0.253	0.935	0.935
Monensin sodium	0.254	0.264	0.372
Dipyrithione	0.263	0.743	0.590
Romidepsin	0.264	0.148	0.187
Dicyclopentamethylenethiuram	0.274	0.743	0.743
Methylene blue	0.307	0.935	0.526
Quinine hemisulfate	0.345	0.235	0.083
Withaferin A	0.372	1.329	0.372

YM155	0.372	0.304	0.526
CyPPA	0.469	0.743	0.590
Adriamycin	0.526	0.935	1.049
1,10-Phenanthroline	0.743	1.177	1.321
AZD-1152-HQPA	0.743	1.482	1.482
CAY10581	0.743	0.662	2.349
Plicamycin	0.833	2.957	2.349
Auranofin	0.935	1.049	1.177
Ruthenium red	0.935	0.264	0.526
Afatinib	0.935	4.176	2.957
GSK-458	0.935	0.332	1.482
Puromycin	1.049	1.482	2.635
Primaquine diphosphate	1.262	0.679	1.077
Clotrimazole	1.866	1.482	1.482
Pyronaridine	1.866	2.635	6.619
Calcimycin	2.635	5.899	1.663
Cyclosporin A	3.317	0.935	1.866
Torin-1	6.619	3.722	5.258
Nizofenone	23.485	14.818	16.626

Note: Each compound was examined in 11 concentrations at a 1:3 dilution for three times against 3D7, HB3 or Dd2 gametocytes. Compounds showing more than 5-fold selectivity in two or three independent experiments against different strains were highlighted.

SI Table 8. Predicted Torin 2 interacting proteins in gametocytes by mass spectrometry experiment.

Protein	ID of *P. falciparum* gene	Molecular weight	Unique peptides
Phosphoribosylpyrophosphate synthetase(Ribose-phosphate diphosphokinase)	PF3D7_1325100	49 kDa	4
Flavoprotein subunit of succinate dehydrogenase (SDHA)	PF3D7_1034400	71 kDa	3
6-phosphofructokinase (PFK11)	PF3D7_1128300	184 kDa	3
RNA-binding protein Nova-1, putative	PF3D7_1415300	38 kDa	3
Deoxyribodipyrimidine photolyase (photoreactivating enzyme, DNA photolyase), putative	PF3D7_0513600	129 kDa	3
Transporter, putative	PF3D7_0914700	58 kDa	3
Conserved Plasmodium protein, unknown function	PF3D7_1036900	193 kDa	3
Heat shock protein 60 (HSP60)	PF3D7_1015600	63 kDa	2
Nuclear protein localization protein 4, putative (NPL4)	PF3D7_0507700	63 kDa	2
Conserved Plasmodium protein, unknown function	PF3D7_1012900	44 kDa	2
Polyadenylate-binding protein, putative	PF3D7_1360900	45 kDa	2
Phosphatase, putative	PF3D7_1464600	170 kDa	2
Rhoptry-associated protein 2 (RAP2)	PF3D7_0501600	47 kDa	2
Deoxyribodipyrimidine photolyase (photoreactivating enzyme, DNA photolyase), putative	PF3D7_0513600	129 kDa	2
Multidrug resistance protein (MDR1)	PF3D7_0523000	162 kDa	2
Rifin (RIF)	PF3D7_0632700	42 kDa	2
Sin3 associated polypeptide p18-like protein	PF3D7_0711400	88 kDa	2
Merozoite surface protein 1 (MSP1)	PF3D7_0930300	196 kDa	2
Glycoprotease, putative	PF3D7_1030600	70 kDa	2
Conserved Plasmodium protein, unknown function	PF3D7_1142800	35 kDa	2
Plasmodium exported protein (PHISTc), unknown function (GEXP12)	PF3D7_1148700	44 kDa	2
Conserved Plasmodium protein, unknown function	PF3D7_1208900	167 kDa	2
DEAD/DEAH box ATP-dependent RNA helicase, putative	PF3D7_1251500	83 kDa	2
Aspartate carbamoyltransferase (atcasE)	PF3D7_1344800	43 kDa	2
Conserved Plasmodium protein, unknown function	PF3D7_1349600	36 kDa	2
Alanyl-tRNA synthetase,Alanine--tRNA ligase (AlaRS)	PF3D7_1367700	165 kDa	2
RNA binding protein, putative	PF3D7_1454000	59 kDa	2
Conserved Plasmodium membrane protein, unknown function	PF3D7_1474600	46 kDa	2
Plasmodium exported protein (PHISTb), unknown function	PF3D7_0402100	68 kDa	1
60S ribosomal protein L4, putative	PF3D7_0507100	46 kDa	1
ATP synthase subunit beta, mitochondrial	PF3D7_1235700	58 kDa	1

Note: Protein bands in both positive (Torin 2 pull-down) and negative (Torin 1 pull-down) samples were destained, reduced, and digested for mass spectrum. The mass spectrum data were analyzed by SEQUEST using PlasmoDB genomic database(http://www.plasmodb.org). Proteins with more than 1 unique peptide in positive samples and 0 unique peptide in negative samples were considered as Torin 2 selective interacting proteins.

SI Fig. 1a, Identifications of phosphoribosylpyrophosphate synthetase, aspartate carbamoyltransferase and transporter as potential targets of Torin 2 by mass spectrometry experiment. Protein bands were destained in a 1:1 mixture of $K_3Fe(CN)_6$ (30 mM) and $Na_2S_2O_3$ (100 mM), dehydrated in acetonitrile, reduced with DTT (50 mM), alkylated by iodoacetamide (120 mM), and digested with sequencing grade trypsin overnight. Digested peptides were extracted with 1% formic acid and subjected to tandem LC-MS/MS analysis. Tandem MS data were used to search PlasmoDB genomic database (http://www.plasmodb.org) using SEQUEST. XCorr is the Sequest value for cross-correlation where values > 2.0 are usually considered as a good correlation. ΔCn is the delta correlation value where values ≥ 0.1 are acceptable.

SI Fig. 1b, A representative mass spectrum of peptide VPVDNLEAQLIGLDYFTK. Tandem MS spectrum of VPVDNLEAQLIGLDYFTK (Phosphoribosylpyrophosphate synthetase) visualized and annotated using scaffold software.

SI Fig. 2, Charaterization of Torin 1 in optic microscopic gametocyte assay and SYBR green asexual parasite assay. (A) Concentration-response curves of Torin 2 and Torin 1 in the optic microscopic gametocyte assay. (B) Concentration-response curves of Torin 2 and Torin 1 in the SYBR green asexual parasite assay.

SI Fig. 3, Synthesis of the positive and negative affinity matrices T2M and T1M. Torin 1 (1-[4-[4-(1-Oxopropyl)-1-piperazinyl]-3-(trifluoromethyl)phenyl]-9-(3-quinolinyl)-benzo[*h*]-1,6-naphthyridin-2(1*H*)-one) and Torin 2 (9-(6-Amino-3-pyridinyl)-1-[3-(trifluoromethyl)phenyl]-benzo[*h*]-1,6-naphthyridin-2(1*H*)-one) were purchased from Tocris. **8c**, **10a** and **10b** were synthesized using the reaction sequence described.

General materials and methods: All commercially available reagents and solvents were purchased and used without further purification. Column chromatography on silica gel was performed on Biotage KPSil pre-packed cartridges using the Biotage SP-1 automated chromatography system. Reverse phase column chromatography was performed on RediSep preparative C-18 column using the Teledyne ISCO combiflash Rf system. ^{1}H spectra were recorded using an Inova 400 MHz spectrometer (Varian). Samples were analyzed on an Agilent 1200 series LC/MS equipped with a Zorbax™ Eclipse XDB-C18 reverse phase (5 micron, 4.6 x 150 mm) column having a flow rate of 1.1 mL/min. The mobile phase was a mixture of acetonitrile and H_2O each containing 0.05% trifluoroacetic acid. A gradient of 5% to 100% acetonitrile over 8 minutes was used during analytical analysis.

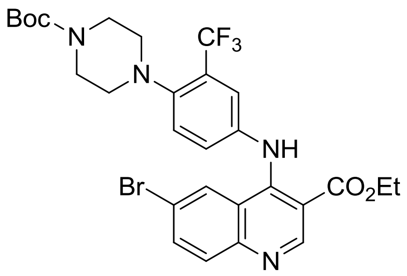

Ethyl 6-bromo-4-((4-(4-(tert-butoxycarbonyl)piperazin-1-yl)-3-(trifluoromethyl)phenyl)amino)quinoline-3-carboxylate (3)

A mixture of *tert*-butyl 4-(4-amino-2-(trifluoromethyl)phenyl)piperazine-1-carboxylate (**2**, 0.691 g, 2.00 mmol) and ethyl 6-bromo-4-chloroquinoline-3-carboxylate (**1,** 0.629 g, 2.00 mmol) in 20 mL of THF was heated in a microwave for 15 min at 120 ^{o}C. The reaction mixture was poured into 50 mL of EtOAc. The solution was washed twice with NaOH solution (1 N, 2x30 mL), dried over $MgSO_4$, filtered and concentrated. The crude product was purified by column chromatography on silica gel using 7-60% EtOAc in hexanes as eluent to give **3** (0.935 g, 75.0%) as a solid. ^{1}H NMR (400 MHz, CHLOROFORM-*d*) δ ppm 10.50 (s, 1 H), 9.28 (s, 1 H), 7.89 (d, *J*=9.00 Hz, 1 H), 7.72 (dd, *J*=9.00, 1.96 Hz, 1 H), 7.63 (d, *J*=2.35 Hz, 1 H), 7.36 (d, *J*=2.35 Hz, 1 H), 7.28 (d, *J*=9.00 Hz, 1 H), 7.14 (dd, *J*=8.61, 2.35 Hz, 1 H), 4.46 (q, *J*=7.30 Hz, 2 H), 3.53 - 3.62 (m, 4 H), 2.83 -2.91 (m, 4 H), 1.49 (s, 9 H), 1.47 (t, *J*=7.30 Hz, 3 H); LC/MS: (electrospray +ve), *m*/*z* 623.1 $(MH)^+$, t_R = 5.90 min, UV_{254} = 100%.

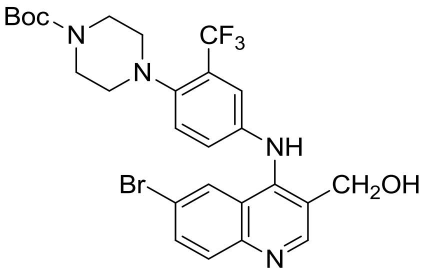

tert-Butyl 4-(4-((6-bromo-3-(hydroxymethyl)quinolin-4-yl)amino)-2-(trifluoromethyl)phenyl)piperazine-1-carboxylate (4)

To a solution of ethyl 6-bromo-4-((4-(4-(*tert*-butoxycarbonyl)piperazin-1-yl)-3-(trifluoromethyl)phenyl)amino)quinoline-3-carboxylate (**3,** 1.24 g, 1.99 mmol) in 200 mL of ethanol was added $NaBH_4$ (0.752 g, 19.9 mmol) at room temperature. After stirring for 4 h, the mixture was poured into 300 mL of EtOAc. The solution was washed with water (3x200 mL). The organic layer was dried over $MgSO_4$, filtered, and concentrated. The residue was purified by column chromatography through a C18 column using 5-100% ACN (containing 0.1% TFA)/water (containing 0.1% TFA) as eluent. The combined pure fractions were neutralized using 1 N NaOH solution, extracted with ethyl acetate (3x50 mL). The organic layer was dried over $MgSO_4$, filtered, and concentrated to give **4** (342 mg, 0.59 mmol, 30%). ^{1}H NMR (400 MHz, DMSO-d_6) δ 9.01 (s, 1H), 8.74 (s, 1H), 8.22 (d, *J* = 2.2 Hz, 1H), 7.96 (d, *J* = 8.9 Hz, 1H), 7.84 (dd, *J* = 8.9, 2.2 Hz, 1H), 7.40 (d, *J* = 8.7 Hz, 1H), 7.03 (d, *J* = 2.7 Hz, 1H), 6.80 (dd, *J* = 8.7, 2.7 Hz, 1H), 5.41 (t, *J* = 5.4 Hz, 1H), 4.43 (d, *J* = 5.5 Hz, 2H), 3.28-3.36 (m, 4H), 2.70-2.76 (m, 4H), 1.41 (s, 9H); LC/MS: (electrospray +ve), *m*/*z* 581.1 $(MH)^+$, t_R = 5.23 min, UV_{254} > 95%.

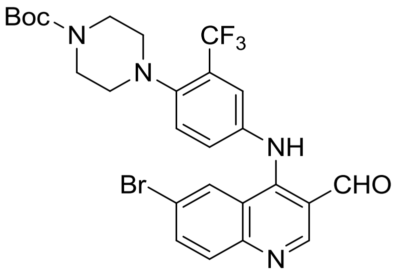

tert-Butyl 4-(4-((6-bromo-3-formylquinolin-4-yl)amino)-2-(trifluoromethyl)phenyl)piperazine-1-carboxylate (5)

To a solution of tert-butyl 4-(4-((6-bromo-3-(hydroxymethyl)quinolin-4-yl)amino)-2-(trifluoromethyl)phenyl)piperazine-1-carboxylate (**4**, 335 mg, 0.576 mmol) in 30 mL of DCM was added Dess-Martin reagent (367 mg, 0.864 mmol). After stirring at room temperature for 2 h, the mixture was poured into 100 mL of ethyl acetate, washed with NaOH solution (1.0 N, 3x50 mL). The organic layer was dried over $MgSO_4$, filtered, and concentrated. The crude product was purified on silica gel using 2-10% MeOH in DCM as eluent to give **5** (257 mg, 77 %) as a solid. ^{1}H NMR (400 MHz, DMSO-d_6) δ 10.38 (s, 1H), 10.01 (s, 1H), 8.91 (s, 1H), 8.16 (d, J = 2.1 Hz, 1H), 7.96-7.83 (m, 2H), 7.57-7.46 (m, 2H), 7.40 (dd, J = 8.7, 2.6 Hz, 1H), 3.40-3.46 (m, 4H), 2.78-2.82 (m, 4H), 1.42 (s, 9H); LC/MS: (electrospray +ve), *m/z* 579.1 $(MH)^+$, t_R = 5.54 min, UV_{254} = 98%.

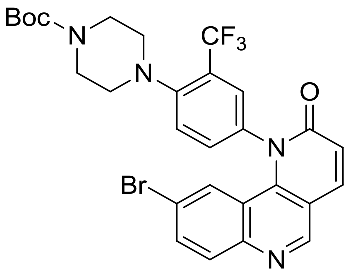

***tert*-Butyl 4-(4-(9-bromo-2-oxobenzo[h][1,6]naphthyridin-1(2H)-yl)-2-(trifluoromethyl)phenyl)piperazine-1-carboxylate (6)**

To a solution of *tert*-butyl 4-(4-((6-bromo-3-formylquinolin-4-yl)amino)-2-(trifluoromethyl)phenyl)piperazine-1-carboxylate (**5**, 251 mg, 0.434 mmol) and ethyl triethyl phosphonoacetate (146 mg, 0.650 mmol) in 3 mL of EtOH was added potassium carbonate (299 mg, 2.166 mmol). After heating at 150 ^{o}C for 15 min in a microwave, the reaction mixture was poured into 50 mL of EtOAc. The solution was washed with NaOH solution (1.0 N, 3x30 mL). The organic layer was dried over $MgSO_4$, filtered, and concentrated. The crude product was purified by column chromatography on silica gel using 2-10% MeOH in DCM as eluent to give **6**

(205 mg, 0.340 mmol, 78 %) as a solid. ^{1}H NMR (400 MHz, DMSO-d_6) δ 9.18 (s, 1H), 8.32 (d, *J* = 9.5 Hz, 1H), 8.00-7.84 (m, 3H), 7.76-7.83 (m, 2H), 6.96 (d, *J* = 9.4 Hz, 1H), 6.54 (d, *J* = 2.1 Hz, 1H), 3.48-3.53 (m, 4H), 2.96-3.32 (m, 4H), 1.44 (s, 9H); LC/MS: (electrospray +ve), *m/z* 603.1 $(MH)^+$, t_R = 6.46 min, UV_{254} = 98%.

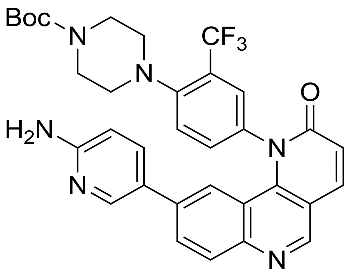

***tert*-Butyl 4-(4-(9-(6-aminopyridin-3-yl)-2-oxobenzo[h][1,6]naphthyridin-1(2H)-yl)-2-(trifluoromethyl)phenyl)piperazine-1-carboxylate (7a)**

A mixture of 5-(4,4,5,5-tetramethyl-1,3,2-dioxaborolan-2-yl)pyridin-2-amine (58.4 mg, 0.265 mmol), *tert*-butyl 4-(4-(9-bromo-2-oxobenzo[h][1,6]naphthyridin-1(2H)-yl)-2-(trifluoromethyl)phenyl)piperazine-1-carboxylate (**6**, 80.1 mg, 0.133 mmol) and $Pd(Ph_3P)_4$ (15.3 mg, 0.013 mmol) in 3 mL of DMF and 0.6 mL of saturated Na_2CO_3 aqueous solution was heated in a microwave for 10 min at 150 ^{o}C. The reaction mixture was filtered through a plug of celite and the filtrate was purified by column chromatography through C18 column using 5-100% ACN (containing 0.1% TFA)/water (containing 0.1% TFA) as eluent. The combined pure fractions were neutralized using 1 N NaOH solution, extracted with ethyl acetate (3x50 mL). The organic layer was dried over $MgSO_4$, filtered, and concentrated to give **7a** (64.2 mg 78%). ^{1}H NMR (400 MHz, DMSO-d_6) δ 9.09 (s, 1H), 8.30 (d, *J* = 9.5 Hz, 1H), 8.05 (d, *J* = 8.7 Hz, 1H), 8.00-7.96 (m, 2H), 7.81-7.67 (m, 3H), 7.10 (dd, *J* = 8.6, 2.6 Hz, 1H), 6.94 (d, *J* = 1.9 Hz, 1H), 6.91 (d, *J* = 9.4 Hz, 1H), 6.41 (dd, *J* = 8.6, 0.8 Hz, 1H), 6.18 (s, 2H), 3.47-3.53 (m, 4H), 2.86-

2.94 (m, 4H), 1.45 (s, 9H). LC/MS: (electrospray +ve), *m/z* 617.2 $(MH)^+$, t_R = 4.63 min, UV_{254} = 95%.

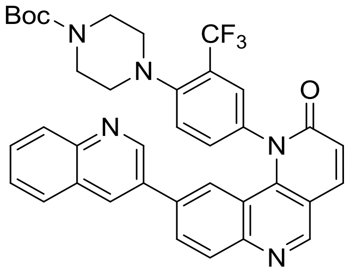

***tert*-Butyl 4-(4-((6-bromo-3-formylquinolin-4-yl)amino)-2-(trifluoromethyl)phenyl)piperazine-1-carboxylate (7b)**

A mixture of 3-quinolineboronic acid (46.2 mg, 0.267 mmol), *tert*-butyl 4-(4-(9-bromo-2-oxobenzo[h][1,6]naphthyridin-1(2H)-yl)-2-(trifluoromethyl)phenyl)piperazine-1-carboxylate (**6**, 80.5 mg, 0.133 mmol) and $Pd(Ph_3P)_4$ (15.4 mg, 0.013 mmol) in 3 mL of DMF and 0.6 mL of saturated Na_2CO_3 aqueous solution was heated in a microwave for 10 min at 150 ^oC. The reaction mixture was filtered through a plug of celite and the filtrate was purified by column chromatography through C18 column using 5-100% ACN (containing 0.1% TFA)/water (containing 0.1% TFA) as eluent. The combined pure fractions were neutralized using 1 N NaOH solution, extracted with ethyl acetate (3x50 mL). The organic layer was dried over $MgSO_4$, filtered, and concentrated to give **7b** (56.8 mg 65.3%). ^1H NMR (400 MHz, DMSO-d_6) δ 9.25 (s, 1H), 8.61-8.66 (m, 1H), 8.40 (d, *J* = 9.5 Hz, 1H), 8.34-8.14 (m, 3H), 8.10-7.99 (m, 3H), 7.90-7.69 (m, 4H), 7.13 (d, *J* = 1.8 Hz, 1H), 7.01 (d, *J* = 9.4 Hz, 1H), 3.33-3.38 (m, 2H), 3.21-3.26 (m, 2H), 2.67-2.56 (m, 2H), 2.46-2.52 (m, 2H), 1.47 (s, 9H); LC/MS: (electrospray +ve), *m/z* 652.2 $(MH)^+$, t_R = 5.96 min, UV_{254} = 95%.

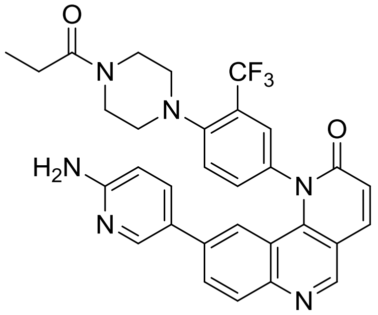

tert-Butyl 4-(4-((6-bromo-3-formylquinolin-4-yl)amino)-2-(trifluoromethyl)phenyl)piperazine-1-carboxylate (8c, WWH30)

To a solution of *tert*-butyl 4-(4-(9-(6-aminopyridin-3-yl)-2-oxobenzo[h][1,6]naphthyridin-1(2H)-yl)-2-(trifluoromethyl)phenyl)piperazine-1-carboxylate (**7a**, 23.5 mg, 0.038 mmol**)** in 3 mL of dichloroethane was added 1 mL of TFA. The mixture was stirred at room temperature for 2 h. The solvent was removed and the residue was dissolved in 3 mL of MeOH. To this solution was added triethylamine (19.3 mg, 0.191 mmol) and propionyl chloride (7.1 mg, 0.076 mmol). The resulted mixture was stirred at room temperature for 4 h. The crude mixture was purified by column chromatography through C18 column using 5-100% ACN (containing 0.1% TFA)/water (containing 0.1% TFA) as eluent. The combined pure fractions were neutralized using 1 N NaOH solution, extracted with ethyl acetate (3x50 mL). The organic layer was dried over $MgSO_4$, filtered, and concentrated to give **8c** (16.7 mg, 77%). ^{1}H NMR (400 MHz, DMSO-d_6) δ 9.19 (s, 1H), 8.34 (d, *J* = 9.5 Hz, 1H), 8.13 (d, *J* = 8.7 Hz, 1H), 8.07 (d, *J* = 2.2 Hz, 1H), 8.03-7.93 (m, 4H), 7.78-7.68 (m, 2H), 7.37 (dd, *J* = 9.3, 2.3 Hz, 1H), 7.00-6.84 (m, 3H), 3.63-3.80 (m, 2H), 3.47-3.57 (m, 2H), 2.87-3.00 (m, 2H), 2.83-2.67 (m, 2H), 2.39 (q, *J* = 7.4 Hz, 2H), 1.04 (t, *J* = 7.4 Hz, 3H). LC/MS: (electrospray +ve), *m*/*z* 573.2 $(MH)^+$, t_R = 3.85 min, UV_{254} = 100%.

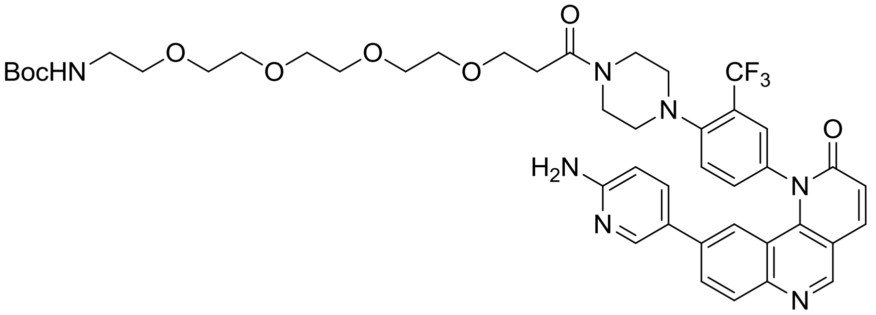

tert-Butyl (15-(4-(4-(9-(6-aminopyridin-3-yl)-2-oxobenzo[h][1,6]naphthyridin-1(2H)-yl)-2-(trifluoromethyl)phenyl)piperazin-1-yl)-15-oxo-3,6,9,12-tetraoxapentadecyl)carbamate (8a)

To a solution of *tert*-butyl 4-(4-(9-(6-aminopyridin-3-yl)-2-oxobenzo[h][1,6]naphthyridin-1(2H)-yl)-2-(trifluoromethyl)phenyl)piperazine-1-carboxylate (**7a**, 57.2 mg, 0.093 mmol) in 3 mL of dichloroethane was added 1 mL of TFA. The mixture was stirred at room temperature for 2 h. The solvent was removed under vacuum and the residue was dissolved in 5 mL of DMF. To this solution was added triethylamine (40 mg, 0.40 mmol), 2,2-dimethyl-4-oxo-3,8,11,14,17-pentaoxa-5-azaicosan-20-oic acid (35.6 mg, 0.097 mmol), and HATU (51.7 mg, 0.111 mmol). The resulted mixture was stirred at room temperature for 6 h. The crude mixture was purified by column chromatography through C18 column using 5-100% ACN (containing 0.1% TFA)/water (containing 0.1% TFA) as eluent. The combined pure fractions were neutralized using 1 N NaOH solution, extracted with ethyl acetate (3x50 mL). The organic layer was dried over $MgSO_4$, filtered, and concentrated to give **8a** (46.1 mg, 58%). ^{1}H NMR (400 MHz, DMSO-d_6) δ 9.09 (s, 1H), 8.30 (d, J = 9.4 Hz, 1H), 8.05 (d, J = 8.7 Hz, 1H), 7.99-7.87 (m, 2H), 7.79-7.69 (m, 3H), 7.14 (dd, J = 8.6, 2.6 Hz, 1H), 6.98-6.88 (m, 2H), 6.74 (t, J = 5.9 Hz, 1H), 6.42 (dd, J = 8.6, 0.8 Hz, 1H), 6.19 (s, 2H), 3.61-3.70 (m, 5H), 3.45-3.55 (m, 10H), 3.23-3.38 (m, 5H), 2.87-3.07

(m, 5H), 2.63-2.70 (m, 3H), 1.36 (s, 9H); LC/MS: (electrospray +ve), *m/z* 864.3 $(MH)^+$, t_R = 4.40 min, UV_{254} = 100%.

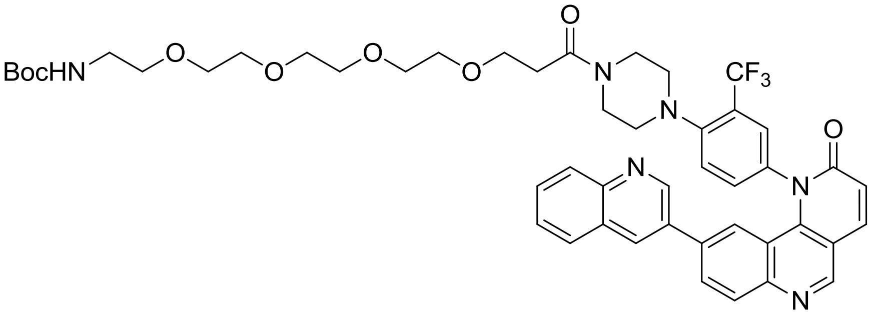

***tert*-Butyl (15-oxo-15-(4-(4-(2-oxo-9-(quinolin-3-yl)benzo[h][1,6]naphthyridin-1(2H)-yl)-2-(trifluoromethyl)phenyl)piperazin-1-yl)-3,6,9,12-tetraoxapentadecyl)carbamate (8b)**

To a solution of *tert*-butyl 4-(4-((6-bromo-3-formylquinolin-4-yl)amino)-2-(trifluoromethyl)phenyl)piperazine-1-carboxylate (**7b**, 50.6 mg, 0.078 mmol**)** in 3 mL of dichloroethane was added 1 mL of TFA. The mixture was stirred at room temperature for 2 h. The solvent was removed under vacuum and the residue was dissolved in 5 mL of DMF. To this solution was added triethylamine (19.3 mg, 0.191 mmol), 2,2-dimethyl-4-oxo-3,8,11,14,17-pentaoxa-5-azaicosan-20-oic acid (29.8 mg, 0.082 mmol), and HATU (35.4 mg, 0.093 mmol). The resulted mixture was stirred at room temperature for 6 h. The crude mixture was purified by column chromatography through C18 column using 5-100% ACN (containing 0.1% TFA)/water (containing 0.1% TFA) as eluent. The combined pure fractions were neutralized using 1 N NaOH solution, extracted with ethyl acetate (3x50 mL). The organic layer was dried over $MgSO_4$, filtered, and concentrated to give **8a** (42.2 mg, 60%). ^1H NMR (400 MHz, DMSO-d_6) δ 9.21 (s, 1H), 8.59 (d, *J* = 2.3 Hz, 1H), 8.36 (d, *J* = 9.5 Hz, 1H), 8.28 (dd, *J* = 2.3, 0.8 Hz, 1H),

8.25-8.13 (m, 2H), 8.10-7.96 (m, 3H), 7.63-7.90 (m, 4H), 7.13 (d, J = 1.7 Hz, 1H), 6.97 (d, J = 9.4 Hz, 1H), 6.73 (t, J = 5.5 Hz, 1H), 3.71-3.43 (m, 19H), 3.35 (t, J = 6.0 Hz, 1H), 3.04 (q, J = 6.0 Hz, 2H), 2.67-2.74 (m, 3H), 2.51-2.62 (m, 3H), 1.36 (s, 9H); LC/MS: (electrospray +ve), m/z 899.3 $(MH)^+$, t_R = 5.43 min, UV_{254} >95%.

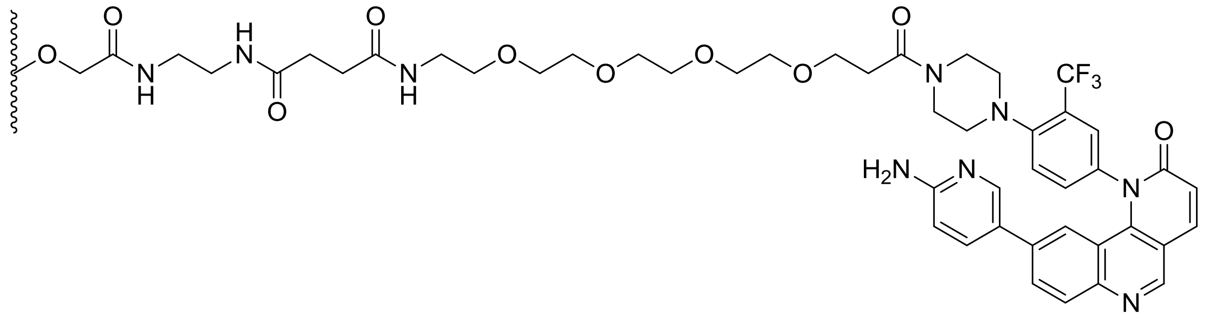

Polymer linked Torin 2 (10a)

To a mixture of Affi-Gel 10 (**9** (Bio-Rad Laboratories, cat. no. 153-6046), 3 mL gel, 45 µmol) and **8a** (12.9 mg, 15 µmole) in 10 mL of DMSO was added triethylamine (150 µmol). The mixture was shaken at room temperature for 6 h (**8a** disappeared from solution based on LC-MS analysis). Then ethanolamine (300 µmol) was added and the resulted mixture was shaken at room temperature for overnight. After washing with DMSO and PBS, the polymer linked Torin 2 (**10a**) was stored in PBS (containing 0.1% sodium azide) at 4 ^{o}C.

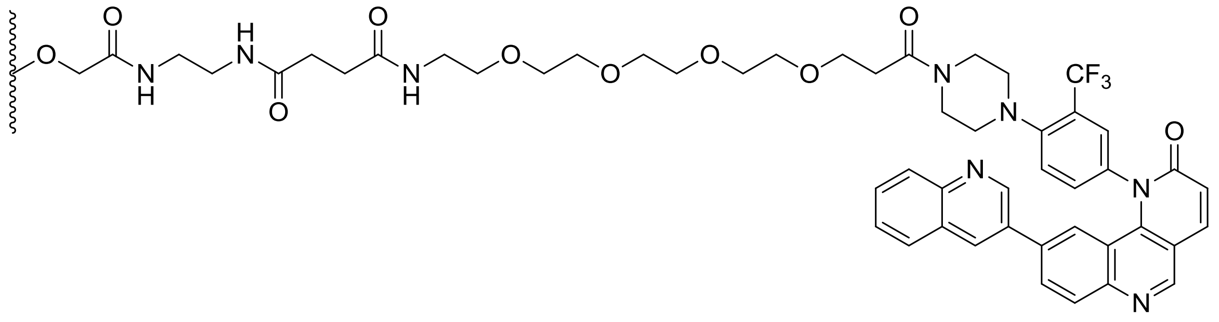

Polymer linked Torin 1 (10b) was prepared in a similar manner as polymer linked Torin 2.

References for Table 1:

For Table 1, Compounds with previously reported activities against asexual parasites[4-23]. Compounds with previously reported activities against gametocytes[4,5,19-21,23].

1 Shevchenko, A., Wilm, M., Vorm, O. & Mann, M. Mass spectrometric sequencing of proteins silver-stained polyacrylamide gels. *Anal Chem* **68**, 850-858 (1996).

2 Ye, X. Y. *et al.* Optimization of protein solubilization for the analysis of the CD14 human monocyte membrane proteome using LC-MS/MS. *J Proteomics* **73**, 112-122 (2009).

3 Anthony, M. P., Burrows, J. N., Duparc, S., Moehrle, J. J. & Wells, T. N. The global pipeline of new medicines for the control and elimination of malaria. *Malar J* **11**, 316 (2012).

4 Buchholz, K. *et al.* A high-throughput screen targeting malaria transmission stages opens new avenues for drug development. *J Infect Dis* **203**, 1445-1453 (2011).

5 Dechy-Cabaret, O. & Benoit-Vical, F. Effects of antimalarial molecules on the gametocyte stage of Plasmodium falciparum: the debate. *J Med Chem* **55**, 10328-10344 (2012).

6 Yuan, J. *et al.* Chemical genomic profiling for antimalarial therapies, response signatures, and molecular targets. *Science* **333**, 724-729 (2011).

7 Fink, E. & Goldenberg, D. M. Antimalarial activity of actinomycin D and cyclophosphamide. *Proc Soc Exp Biol Med* **132**, 165-167 (1969).

8 Greif, G., Harder, A. & Haberkorn, A. Chemotherapeutic approaches to protozoa: Coccidiae - current level of knowledge and outlook. *Parasitol Res* **87**, 973-975 (2001).

9 Crowther, G. J. *et al.* Identification of inhibitors for putative malaria drug targets among novel antimalarial compounds. *Mol Biochem Parasit* **175**, 21-29 (2011).

10 Mehlhorn, H., Ganster, H. J. & Raether, W. Effect of Salinomycin-Na on Malaria Parasites (Plasmodium-Falciparum and P-Berghei). *Zbl Bakt-Int J Med M* **256**, 305-313 (1984).

11 Hoppe, H. C. *et al.* Antimalarial quinolines and artemisinin inhibit endocytosis in Plasmodium falciparum. *Antimicrob Agents Ch* **48**, 2370-2378 (2004).

12 Ginsburg, H., Nissani, E., Krugliak, M. & Williamson, D. H. Selective Toxicity to Malaria Parasites by Non-Intercalating DNA-Binding Ligands. *Mol Biochem Parasit* **58**, 7-15 (1993).

13 Sannella, A. R. *et al.* New uses for old drugs. Auranofin, a clinically established antiarthritic metallodrug, exhibits potent antimalarial effects in vitro: Mechanistic and pharmacological implications. *Febs Lett* **582**, 844-847 (2008).

14 Ekong, R. M., Kirby, G. C., Patel, G., Phillipson, J. D. & Warhurst, D. C. Comparison of the in vitro activities of quassinoids with activity against Plasmodium falciparum, anisomycin and some other inhibitors of eukaryotic protein synthesis. *Biochem Pharmacol* **40**, 297-301 (1990).

15 Yapi, A. D. *et al.* In vitro and in vivo antimalarial activity of derivatives of 1,10-phenanthroline framework. *Arch Pharm (Weinheim)* **339**, 201-206 (2006).

16 Krogstad, D. J. *et al.* Calcium and the malaria parasite: parasite maturation and the loss of red cell deformability. *Blood Cells* **17**, 229-241; discussion 242-228 (1991).

17 Bobbala, D. *et al.* Effect of cyclosporine on parasitemia and survival of Plasmodium berghei infected mice. *Biochem Biophys Res Commun* **376**, 494-498 (2008).

18 Tanaka, T. Q. & Williamson, K. C. A malaria gametocytocidal assay using oxidoreduction indicator, alamarBlue. *Mol Biochem Parasit* **177**, 160-163 (2011).

19 Hanson, K. K. *et al.* Torins are potent antimalarials that block replenishment of Plasmodium liver stage parasitophorous vacuole membrane proteins. *Proc Natl Acad Sci U S A* (2013).

20 Li, H. *et al.* Validation of the Proteasome as a Therapeutic Target in Plasmodium Using an Epoxyketone Inhibitor with Parasite-Specific Toxicity. *Chem Biol* **19**, 1535-1545 (2012).
21 Toye, P. J., Sinden, R. E. & Canning, E. U. The action of metabolic inhibitors on microgametogenesis in Plasmodium yoelii nigeriensis. *Z Parasitenkd* **53**, 133-141 (1977).
22 Chong, C. R., Chen, X. C., Shi, L. R., O Liu, J. & Sullivan, D. J. A clinical drug library screen identifies astemizole as an antimalarial agent. *Nat Chem Biol* **2**, 415-416 (2006).
23 D'Alessandro, S. *et al.* A Plasmodium falciparum screening assay for anti-gametocyte drugs based on parasite lactate dehydrogenase detection. *J Antimicrob Chemother* (2013).

SI Fig. 1a

Protein name	Peptide sequence	Spectrum charge	XCorr	DCn
Phosphoribosylpyrophosphate synthetase	VPVDNLEAQLIGLDYFTK	2	6.1	0.582
	KDLYKPVIVSPDAGGVYR	3	3.66	0.327
	SPLEEVVVTDTVK	2	4.14	0.435
	VPISAADVAR	2	2.99	0.344
	VPISAADVAR	2	3.11	0.457
Aspartate carbamoyltransferase (atcasE)	VFCSVFLEPSTR	2	2.69	0.141
Transporter, putative	ATGSSMTDK	2	2.64	0.182
	TGAFSELCK	2	2.87	0.179

SI Fig. 1b

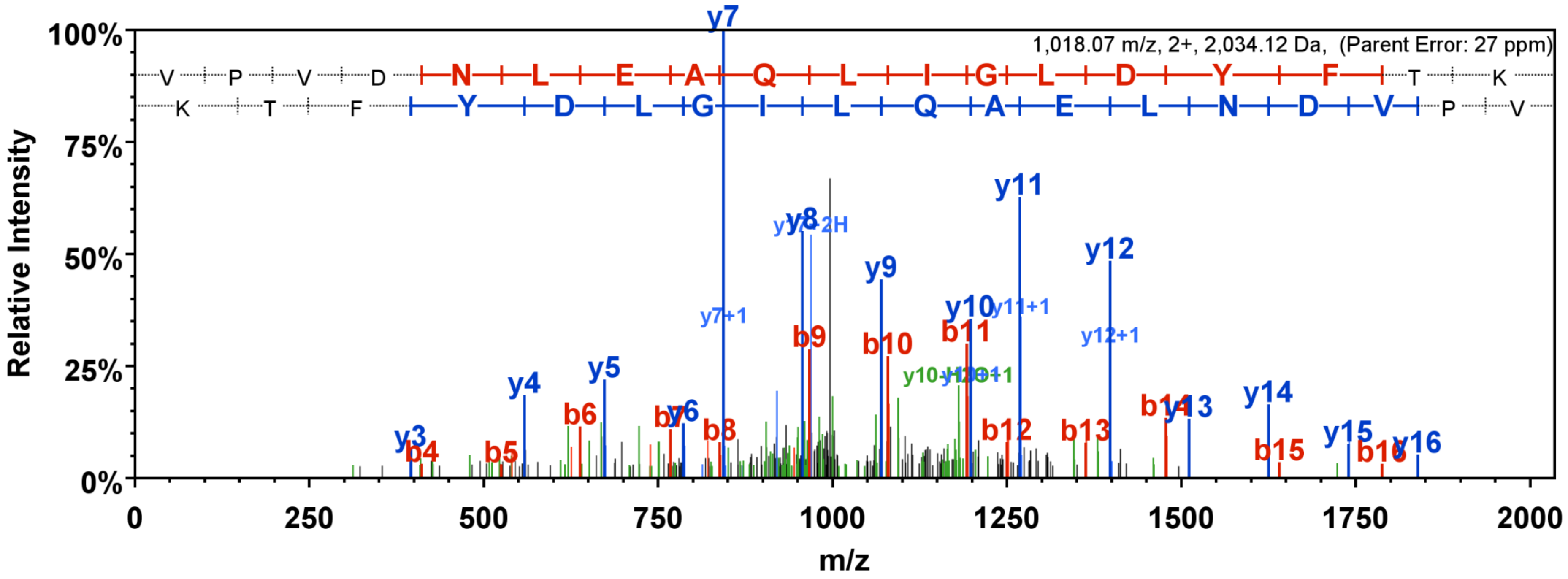

1,018.07 m/z, 2+, 2,034.12 Da, (Parent Error: 27 ppm)
V P V D N L E A Q L I G L D Y F T K
K T F Y D L G I L Q A E L N D V P V
Relative Intensity
100%
75%
50%
25%
0%
m/z
0
250
500
750
1000
1250
1500
1750
2000
y7
y11
y8
y12
y9
y10
y11+1
y7+1
y12+1
b9
b10
b11
y4
y5
b6
y6
b8
b12
b13
y13
y14
y3
b4
b5
b15
y15
y16

SI Fig. 2

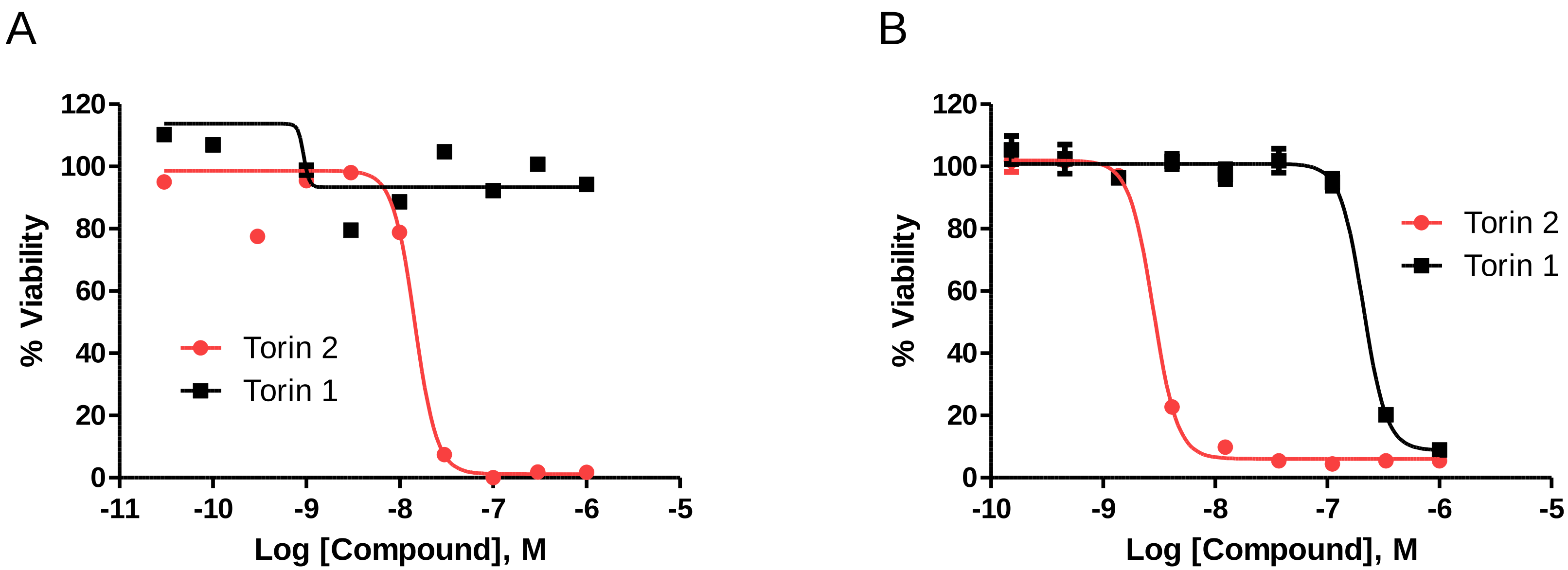
A
120
100
80
60
40
20
0
% Viability
Torin 2
Torin 1
-11
-10
-9
-8
-7
-6
-5
Log [Compound], M
B
120
100
80
60
40
20
0
% Viability
Torin 2
Torin 1
-10
-9
-8
-7
-6
-5
Log [Compound], M

SI Fig. 3

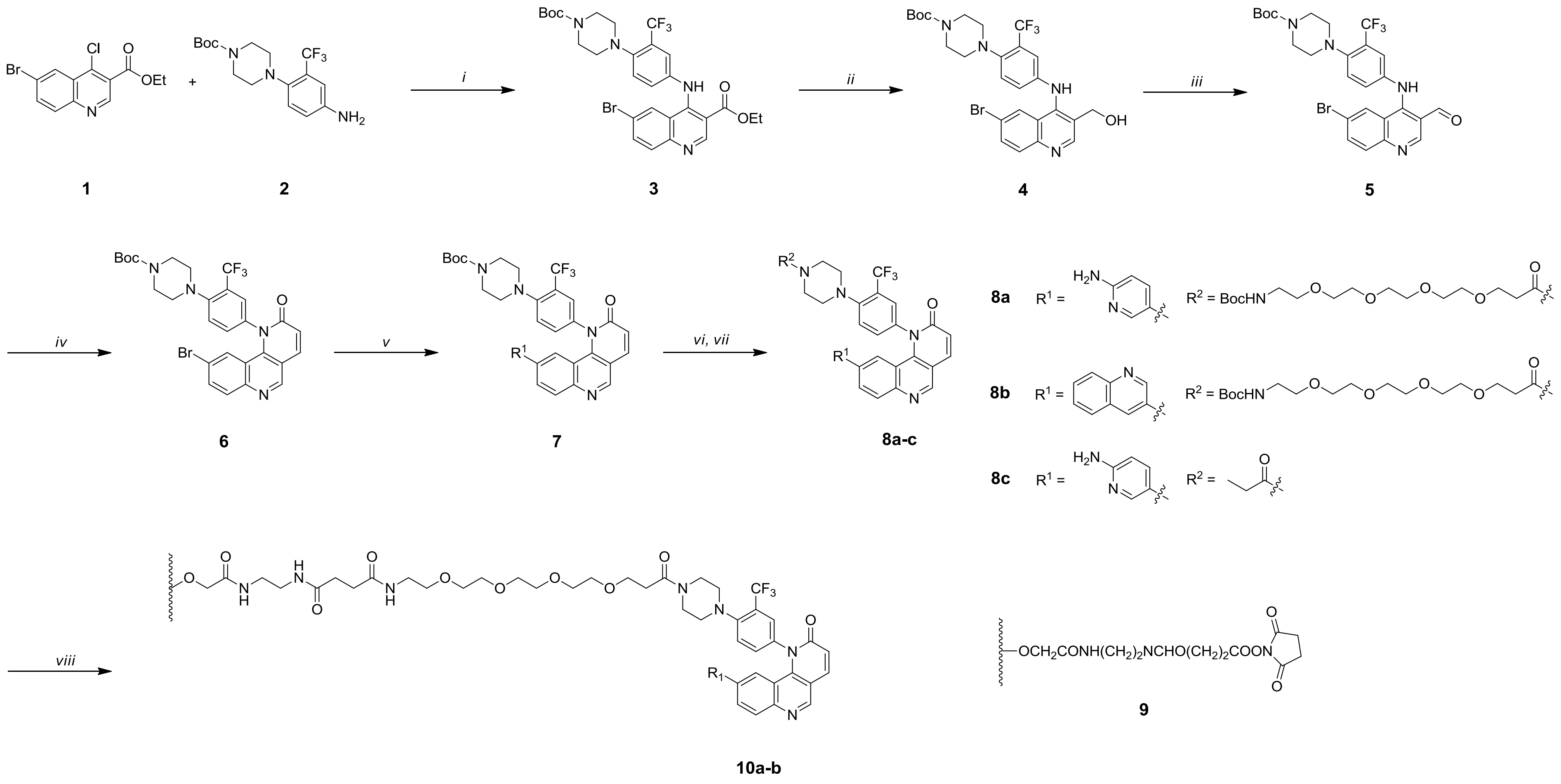

Reagents and reaction conditions: (i) THF, 120 ^oC, MW, 15 min; (ii) EtOH, $NaBH_4$, r.t. 4 h; (iii) DCM, Dess-Martin reagent, r.t., 2 h; (iv) EtOH, triethyl phosphonoacetate, K_2CO_3, 150 ^oC, MW, 15 min; (v) $R^1B(OR')_2$; $Pd(PPh_3)_4$, Na_2CO_3, MW, 150 ^oC, 10 min; (vi) TFA, DCE, r.t. 2 h; (vii) for **8a-b:** R^2OH**,** HATU, Et_3N, DMF, r.t. 6 h; for **8c**:CH_3CH_2COCl, Et_3N, r.t. 4 h; (viii) (a) TFA, DCE; (b) **9**, DMSO, r.t. o/n
